# Pioneering the preparation of porous PIM-1 membranes for enhanced water vapor flow†

**DOI:** 10.1039/d3ra08398e

**Published:** 2024-03-22

**Authors:** Esra Caliskan, Sergey Shishatskiy, Volker Abetz, Volkan Filiz

**Affiliations:** a Institute of Membrane Research, Helmholtz-Zentrum Hereon Max-Planck-Str. 1 Geesthacht 21502 Germany volkan.filiz@hereon.de +49-41-5287-2425; b Institute of Physical Chemistry, University of Hamburg Martin-Luther-King-Platz 6 Hamburg 20146 Germany

## Abstract

In this study, porous polymers of intrinsic microporosity (PIM-1) membranes were prepared by non-solvent induced phase inversion (NIPS) and investigated for water vapor transport in view of their application in membrane distillation (MD). Due to the lack of high boiling point solvents for PIM-1 that are also water miscible, the mixture of tetrahydrofuran (THF) and *N*-methyl-2-pyrrolidone (NMP) was found to be optimal for the formation of a membrane with a developed porous system both on the membrane surface and in the bulk. PIM-1 was synthesized by using low and high temperature methods to observe how molecular weight effects the membrane structure. Low molecular weight PIM-1 was produced at low temperatures, while high molecular weight PIM-1 was obtained at high temperatures. Several membranes were prepared, including PM-6, PM-9, and PM-11 from low molecular weight PIM-1, and PM-13 from high molecular weight PIM-1. Scanning electron microscopy (SEM) was used to image the surface and cross-section of different porous PIM-1 membranes. Among all the PIM-1 membranes (PM) obtained, PM-6, PM-9, PM-11 and PM-13 showed the most developed porous structure, while PM-13 showed large voids in the bulk of the membrane. Contact angle measurements showed that all PIM-1 porous membranes are highly hydrophobic. Liquid water flux measurements showed that PM-6, PM-9 and PM-11 showed minimal water fluxes due to small surface pore size, while PM-13 showed a high water flux due to a large surface pore size. Water vapor transport measurements showed high permeance values for all membranes, demonstrating the applicability of the developed membranes for MD. In addition, a thin film composite (TFC) membrane with PIM-1 selective layer was prepared and investigated for water vapor transport to compare with porous PIM-1 membranes. The TFC membrane showed an approximately 4-fold lower vapor permeance than porous membranes. Based on these results, we postulated that the use of porous PIM-1 membranes could be promising for MD due to their hydrophobic nature and the fact that the porous membranes allow vapor permeability through the membrane but not liquid water. The TFC membrane can be used in cases where the transfer of water-soluble contaminants must be absolutely avoided.

## Abstract 2

In this study, porous membranes of polymers of intrinsic microporosity (PIM-1) were prepared by non-solvent induced phase inversion (NIPS) and investigated for water vapor transport in view of their application in membrane distillation (MD).

## Graphical abstract

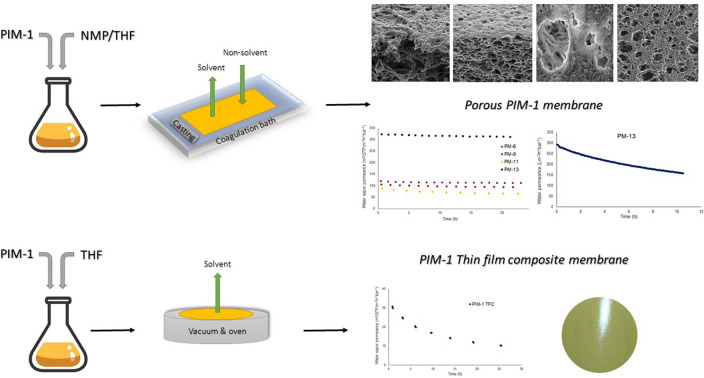

## 1. Introduction

Water scarcity is one of the biggest hurdles that science is trying to overcome today. According to a report published by the United Nations in 2023, it is estimated that the global urban population suffering from water scarcity could reach 1.7 to 2.4 billion by 2050.^1^ Global population growth, industrialization, wars and climate change are making access to water resources more difficult, while freshwater reserves and conventional energy sources are rapidly depleting.^2^ In other words, due to the lack of cost-effective technologies to remove unwanted solutes such as ions, organics and particles, the vast majority of water in natural sources is unsuitable for human consumption or many other industrial uses.^3^ As a result, the development of efficient and sustainable water treatment technologies is the focus of research by many groups around the world.^4^ Membrane processes are among the leading water treatment technologies due to their higher energy efficiency and smaller footprint compared to other available, mostly thermal options.^5,6^ Pressure–driven processes, such as reverse osmosis (RO) and nanofiltration (NF), are the conventional membrane technologies currently in use.^7^ However, the transport of water through the molecular-sized 'pores' of the membrane in both RO and NF technologies requires a significant amount of pressure, which results in high-energy penalties and consequently high operating costs. Despite being a less energy-intensive process than RO, NF has a lower rejection rate for sodium and chloride ions, which are the main dissolved substances in saline water.^8,9^ However, novel membrane technologies that use low-grade energy as the driving force for separation are being extensively studied and developed. One such technology is membrane distillation (MD), which has attracted considerable interest. Conventionally, MD is a thermal-membrane process driven by the vapor pressure difference between hot and cold sides of a hydrophobic porous or non-porous membrane. The vapor pressure difference between the feed and permeate sides of the membrane, and a chemical potential gradient too low for other components, results in only vapor molecules being able to pass across the membrane followed by condensation of the vapor molecules in the cold permeate side of the membrane module.^10,11^ The use of MD has several advantages, including lower operating temperatures compared to conventional processes, which means that the feed solution does not need to be heated to its boiling point. In addition, the feed hydrostatic pressure in MD is significantly lower than in pressure-driven membrane processes such as RO, making it a potentially cost-effective process with lower requirements for membrane mechanical properties.^12,13^ Moreover, the pore size of the membrane used in MD is relatively larger than those for other membrane separation applications which makes MD less affected by fouling.^14^

There are several types of MD configurations:^15,16^

• Direct contact membrane distillation (DCMD) involves direct contact between the hot feed solution and the hot membrane surface. Water vapor passes through the membrane by the gradient of chemical potential between the feed and the permeate sides of the membrane.

• Air gap membrane distillation (AGMD) contains stagnant air gap present between permeate side of the membrane and the condensation surface meanwhile feed solution is in direct contact with the feed side of the membrane.

• Sweeping gas membrane distillation (SGMD) uses a gas stream to sweep the vapor on the permeate side and to drive it to a condenser.

• Vacuum membrane distillation (VMD) employs a pump to create the vacuum on the permeate side in order to enhance the mass transfer rate.

A variety of polymers are used in MD depending on the specific process conditions. Some examples of polymers commonly used for MD membranes include polytetrafluoroethylene (PTFE), poly(vinylidene fluoride) (PVDF) and polypropylene (PP) due to their low surface tension and high hydrophobicity.^17^ The membranes typically have a volume porosity in the range of 0.60–0.95, which is determined by the absence of the need to withstand an absolute pressure gradient across the membrane, while the surface pore size is in the range of 0.2–1.0 μm.^15^ Moreover, hydrophobized ceramic membranes and carbon nanotubes are being used in MD owing to their hydrophobicity and porosity.^18^

There are also several challenges that need to be addressed to make MD more practical and economically viable. Two key factors significantly affect the performance of MD. The first is ‘membrane wetting’, which occurs when water vapor condenses in the pores of the membrane.^19^ The accumulation of condensed water in the membrane pores causes a reduction in permeability, while at the same time changing the surface property of the membrane, making it less hydrophobic and increasing its affinity for water molecules.^20^ The other factor hindering MD performance is ‘membrane fouling’, which is caused by the accumulation of organic or inorganic substances, dissolved or colloidal in the feed water, on the membrane surface or in the pores of the membrane, resulting in a reduction in vapor flux and a breakthrough of the feed mixture to the permeate side.^21,22^ These two main factors limit the choice of a suitable polymer for MD. As mentioned above, the MD process requires hydrophobic and porous membranes in order to restrict all molecules except water from passing through the membrane while avoiding membrane wetting. In addition to conventional polymers such as PTFE, PVDF and PP, the availability of other effective polymers that meet these requirements for MD applications is being investigated.

In the last two decades, there has been considerable interest in polymers of intrinsic microporosity (PIMs), a new class of microporous polymers with many attractive properties such as excellent solubility in organic solvents and thus processability, high glass transition temperature, good thermal stability, and exceptional mechanical and film-forming properties.^23–26^ In addition to these features, PIM-1 acquires two key properties, which are ‘hydrophobicity’ and ‘high free volume’, which can be exploited in favor of MD applications. Although, there is a substantial amount of research on gas separation using PIM-1,^27–29^ there are not many studies specifically focusing on PIM-1 for water separation.

Previous studies on PIM-1 for water separation have been focused on various modifications of this polymer.^30,31^ Kim *et al.*^30^ studied the carbonization of PIM-1 resulting in an increase of water flux in nanofiltration application. Furthermore, Jeon *et al.*^31^ reported on the study of carboxylate-functionalized PIM-1 developed for nanofiltration as well. In addition to these studies, there are some researches focused on the development of PIM-1 hollow fiber membranes, which may be related to the current study in terms of aiming to obtain similar morphological aspects of PIM-1. For example, Jue *et al.*^32^ developed PIM-1 hollow fiber membrane used for gas separation where they attempted to obtain integrally asymmetric membrane by phase inversion. In another study, Hao *et al.*^33^ described the study of ultem/PIM-1 hollow fiber membrane obtained by electrospinning. However, none of these studies were focused on the water separation application of PIM-1 with membranes obtained by NIPS.

Despite the versatile properties of PIM-1, there is still a lack of studies on porous PIM-1 membranes for water separation applications. Considering this knowledge deficit in the literature, the aim of this paper is to describe asymmetric porous PIM-1 membrane supported by a nonwoven for prospective use in membrane distillation. For this purpose, the phase inversion method, which is the most common method of porous membrane formation, was implemented. The choice of solvent and non-solvent is critical for the preparation of porous PIM-1 membranes by NIPS as it can affect the morphology, pore size, and overall performance of the resulting PIM-1 membranes.^34,35^ In general, the solvent should dissolve the polymer and acquire a high boiling point in order not to evaporate during the membrane formation process, while the non-solvent should not dissolve the polymer and induce phase separation. There is an exiguity of solvents for PIM-1 that acquire a high boiling point. For this reason, an attempt was made to use a mixture of tetrahydrofuran (THF) and *N*-methyl-2-pyrrolidone (NMP), although NMP is a weak solvent for PIM-1. Water was preferred as the coagulation bath as it is the most environmentally friendly and widely available liquid. After casting the porous PIM-1 membrane, water vapor and water flux analyses were carried out. In addition, a TFC membrane with a dense PIM-1 selective layer was fabricated, and water vapor transport data was obtained and compared with that of the porous membrane.

## 2. Materials and methods

### 2.1 Materials

5,5′,6,6′,-Tetrahydroxy-3,3,3′,3′-tetramethyl-1,1′-spirobisindane (TTSBI, 98%) was purchased from ABCR GmbH. 2,3,5,6-tetrafluoro-terephthalonitrile (TFTPN, 99%) was purchased from Lanxess. TFTPN was sublimated twice at 70 °C under vacuum prior to use. Potassium carbonate (K_2_CO_3_, 99%) was purchased from Alfa Aesar. The rest of the commercially available compounds as chloroform (CHCl_3_), dichlorobenzene (DCB), dimethylacetamide (DMAc), ethanol (EtOH), NMP, THF were obtained from Merck Millipore and were used without further treatment.

### 2.2 Synthesis of PIM-1

In the current study, PIM-1 was synthesized three times using different methods. The first batch was synthesized according to the high temperature method^36^ to achieve higher polymer molecular weight. The next two batches were synthesized according to the low temperature method^37^ yielding polymers with lower molecular weight. Since the low temperature method ensures more reproducible molecular weight^38^ it has been chosen for the trials on porous membrane formation. In the low temperature method, it is assumed that if the molecular weights of the polymer are close to each other, the properties of the polymer will not change significantly.

The polymers resulted from two synthesis methods had characteristic yellow color. Polymers were characterized by size-exclusion chromatography calibrated to polystyrene standards for apparent average molecular weight (*M*_w_) and dispersity (*Đ*) summarized in Table 1.

**Table 1:** Molecular weight and dispersity of different synthesis of PIM-1

Method	Name of batch	*M*_w_ (kg mol^−1^)	*Đ*
High temperature	PIM-HT	142	5.5
Low temperature	PIM-LT-1	76	4.4
Low temperature	PIM-LT-2	76	2.8

### 2.3 Preparation of membranes

#### 2.3.1 Membrane obtained by non-solvent induced phase separation

The choice of the membrane casting method is critical to the production of polymeric membranes for use in various membrane applications such as gas separation and water treatment. There are several techniques to form polymeric membranes, which can be porous, dense, or porous with a dense thin layer on top. In particular, there are numerous techniques to fabricate porous membranes, such as sintering, stretching, track-etching.^39^ In addition to these techniques, phase inversion is the most widely used method for producing porous membranes due to its simplicity, low cost and scalability.^40^ In this method, a homogeneous polymer solution is first cast onto a nonwoven fabric using a doctor blade to obtain a polymer solution layer of uniform thickness. The resulting solution coated nonwoven is then immersed in a non-solvent or subjected to a rapid temperature change in order to induce phase separation.^41^ If a non-solvent is used, phase separation occurs due to the diffusion of the non-solvent into the thermodynamically stable polymer solution, causing a rapid decay of solvent system's ability to dissolve a polymer with consequent precipitation of a polymer and thus formation of polymer-rich and polymer-poor phases. At the same time, solvents of the casting solution diffuse out of the area of two phases. Basically, the polymer-rich phase forms the membrane matrix, while the polymer-poor phase forms the pores of the membrane.^42^ A high concentration of a volatile solvent in the casting solution can promote the formation of a dense skin layer over an underlying porous structure when the polymer solution is exposed to air after deposition onto nonwoven, allowing some solvent to be evaporated. If a porous membrane is desired, it is necessary to minimize the time the solution is exposed to the air. The composition of the casting solution, especially the amount of highly volatile solvents, the duration of solvent evaporation prior to precipitation, and the type of precipitation bath, its temperature, are crucial parameters for achieving the desired membrane structure.^43,44^

In this study, various solvents were tested in order to prepare a suitable polymer–solvent composition considering the factors mentioned above. Table 2 shows the most suitable polymer composition of the casting solutions used in this study for the non-solvent induced phase inversion method and the casting parameters for membrane formation. On the other hand, other polymer solutions prepared and membranes cast are shown in the ESI, Table S1.†

**Table 2:** Composition of casting solutions and membrane casting parameters

Name	Composition (% wt)	Precipitation bath	Batch	Membrane casting thickness	*M*_w_ (kDa)
PM-6	PIM/NMP/THF:12.5/69.5/18	Water	PIM-LT-2	150 μm	76
PM-9	PIM/NMP/THF:12.5/69.5/18	Water	PIM-LT-2	150 μm	76
PM-11	PIM/NMP/THF:15/77/8	Water	PIM-LT-1	150 μm	76
PM-13	PIM/NMP/THF:11.5/72/16.5	Water	PIM-HT	150 μm	142

The concentration of PIM-1 polymer in the casting solutions was adjusted to be between 10 and 17.5 wt% depending on the solvents used. After stirring for 2 days, the solution was directly cast on a polyester nonwoven support using an in-house designed casting machine with a doctor blade set at a specified gap height (some membranes were cast directly onto glass as substrate, see Table 2). On the casting machine, the speed of the take-up reel is adjustable to control the evaporation time before the solution cast film is immersed in the non-solvent (precipitation) bath. The cast membranes were left in the precipitation bath for 20 min, pulled out of the water and dried under vacuum at 60 °C for 2 days to remove all residual solvents. Due to the lack of high boiling point solvents suitable for PIM-1 dissolution, THF was chosen as a component of all polymer solutions except those with DCB. NMP is one of the most used nonvolatile solvents for membrane formation by NIPS. As NMP is a weak solvent of PIM-1, the combination of NMP and THF was essential and gave the best results among other investigated solvent systems. Chlorinated solvents, as *e.g.*, chloroform, which is one of the best solvents for PIM-1, were considered environmentally harmful and not suitable for potential large-scale membrane production. Another argument against using chlorinated solvents is their immiscibility with water, the most desirable liquid to use as a non-solvent in the quenching bath.

A further treatment was applied to one of the membranes (PM-6) to investigate the permeance of liquid water while the membrane was initially fully wetted with a liquid. For that purpose, isopropanol was used as wetting agent to reduce the contact angle of the membrane toward water. After membrane preparation by phase inversion method, dry PM-6 membrane was immersed in 50 : 50 (wt%) isopropanol–water mixture for 1 hour. Afterwards, the membrane was taken out of the solution and subjected immediately to water flux measurement.

#### 2.3.2 Thin film composite membrane

The preparation of the TFC membrane was carried out by a coating method, which is widely used for the formation of TFC membranes. The porous support was brought into the contact with the polymer solution and drawn from the contact point at a certain speed to achieve uniform polymer solution distribution on the ultrafiltration membrane used as a support.^45^ In this study, a PIM-1 dense selective layer was formed on a porous polyacrylonitrile (PAN) membrane using a laboratory scale membrane casting machine developed in-house. The solution of PIM-1 was prepared as 1% by weight in THF. The polymer solution was poured into the bath of specific shape and the PAN membrane was brought into contact with the solution. A meniscus of polymer solution was then formed by lowering the bath with the solution for *ca.* 2 mm. A thin layer of solution on the support surface was obtained by pulling the porous support out of the meniscus at a constant speed. The drying of the TFC membrane was carried out under ambient conditions without controlling of the solvent evaporation. The procedure was carried out in a hood with a high air exchange rate to ensure that no accountable solvent vapor was present in the vicinity of the membrane casting machine, except where the polymer solution bath was placed.

### 2.4 Characterization methods

#### 2.4.1 Size-exclusion chromatography (SEC)

SEC measurements were performed at 30 °C in CHCl_3_ using a column combination (precolumn-SDV-linear, SDV-linear and SDV 102 nm with inner diameter = 4.6 mm and length = 53 cm, Polymer Standard Service GmbH, Mainz, Germany) at a flow rate 1.0 mL min^−1^. A combination of refractive index (RI) and ultraviolet (UV) detectors was used for concentration detection. The system was calibrated to polystyrene standards for the evaluation of apparent weight average molecular weight (*M*_w_) and dispersity index (*Đ*) of the prepared polymers.

#### 2.4.2 Scanning electron microscopy (SEM)

The morphology of the membranes was studied using a MERLIN SEM (ZEISS, Oberkochen, Germany) at accelerating voltages between 1.5 kV and 3 kV. The samples were coated with 1–1.5 nm platinum. The cross-section morphology was examined on cryogenically (liquid nitrogen) fractured samples.

#### 2.4.3 Fourier transform infrared spectroscopy (FTIR)

FTIR spectra was measured on an ALPHA FTIR spectrometer (Bruker Optics, Bremen, Germany) in attenuated total reflectance mode (ATR, diamond crystal). The measurements were conducted at ambient temperature in a spectral range of 400 to 4000 cm^−1^ with a resolution of 4 cm^−1^ and an average of 64 scans.

#### 2.4.4 Water contact angle

Dynamic contact angle measurements were conducted by using Kruess Drop Shape Analysis System DSA 100. The computational analysis was done using Advance software. Prior to each measurement, the baseline detection was done manually. Deionized water was taken to glass syringe and placed onto PIM-1 samples. Average value of first 30 measurement was recorded for each sample.

#### 2.4.5 Water flux measurement

Water flux measurements were performed at ambient conditions using an in-house designed automatic testing facility where dead-end mode of filtration was utilized. The transmembrane pressure (Δ*p*) was set to 4 bar. This pressure was chosen in order to compare results with parameters obtained for other membranes developed at Hereon. The diameter of the membrane was 1.45 cm (*A* = 1.65 cm^2^). Ultrapure water with conductivity ≤0.055 μS cm^−1^ from Siemens LaboStar was used for this measurement. The volume of water (Δ*V*) permeated through the membrane was determined every 60 s using build-in microbalance (Δ*t*). The normalized permeance (*J*) was calculated according to following equation:1
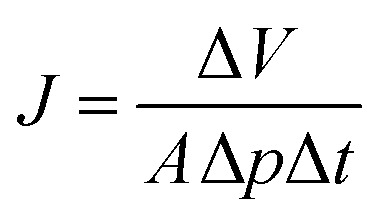
In general, pore wetting should be avoided in order to maintain an efficient MD operation. Membrane wetting is obtained when applied pressure exceeds the liquid entry pressure (LEP) which can be defined as the highest applied transmembrane hydrostatic pressure before the liquid in the feed penetrates the larger pores and passes through the hydrophobic membrane. LEP can be calculated by the Young–Laplace equation as seen in (eqn (2)).^46^2
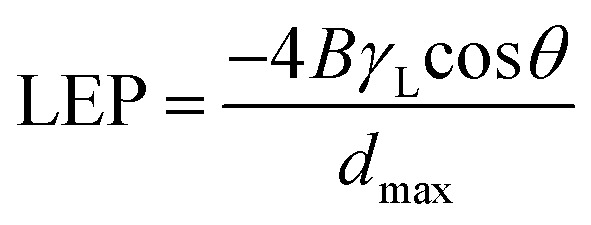
where *B* is the geometric factor of the membrane pores (*B* = 1 for assumed cylindrical pores), *γ*_L_ is surface tension of the liquid, *θ* is the contact angle of the liquid and *d*_max_ the largest pore size. In theory, when transmembrane pressure is kept below the LEP during the operation, the liquid in the feed does not penetrate the pores, thus, only vapor diffusion across the membrane occurs.^47^ LEP for membranes under investigation was assessed during experiment where feed pressure was stepwise increased until constant water flow was detected.

#### 2.4.6 Water vapor permeance test

In the MD water is transported across the membrane in the vapor state, unless there is no liquid breakthrough. It was decided to investigate vapor transport of PIM-1 porous and TFC membranes using an in-house designed pressure increase facility to have parameters comparison to other membranes developed at Hereon. This facility uses the same “constant volume/variable pressure” measurement method as the time-lag facility, which is widely used to determine gas transport parameters of thick isotropic polymer films.^48^ The software of the pressure increase facility is optimized for the characterization of membranes with negligible thickness for which no time-lag can be determined. All membranes were characterized at 40 °C, feed vapor pressure lower than 60 kPa and permeate pressure changing in the range 10–130 Pa. The permeance was determined as the average of at least 10 measurements within each data accumulation set.^49^ The foreseen and accepted disadvantage of the pressure increase facility is the lack of ability to determine vapor permeance at saturated vapor conditions. This disadvantage arises from the fact that the gas or vapor to be studied is accumulated and conditioned to the temperature of the experiment in the feed pressure vessel and is isolated from the external supply line at the start of the experiment. When an experiment starts, the valve on the feed side of the membrane is opened and the membrane is exposed to the penetrant. This results in a small pressure drop, approximately 10% of the pressure at which gas or vapor was accumulated in the feed vessel. At the same time, this method allows the behavior of the membrane to be studied as it is subjected to a steadily decreasing feed pressure of the penetrant. In the case of highly soluble or condensable penetrants, the results of such an experiment are of great importance.

## 3. Results and discussion

### 3.1 Porous membrane development

The aim of this work was to develop porous PIM-1 membranes by NIPS for use in water purification processes, preferably in MD. There is a lack of studies on this subject, as PIM-1 is a difficult polymer to use in the phase inversion process. The solvent choice is crucial for the phase separation method and when the subject is PIM-1, there is a limitation in solvents suitable for this membrane formation process, especially when water is used as phase separation inducing liquid. The most prevalent solvents used to cast PIM-1 membranes or films are THF, CHCl_3_, dichloromethane (DCM) and DCB but not all of them can be used in the phase inversion method because CHCl_3_, DCB and DCM are immiscible with water, and high volatility of CHCl_3_ and DCM can easily induce dense layer formation when polymer solution is exposed to air during membrane casting. Considering these limitations, there are not many options left but to use THF as a solvent. However, it is important to use as much of the high boiling point solvent in the polymer solution that is well miscible with water to facilitate the phase inversion process. With this in mind, we have tried several solvent/non-solvent combinations to prepare polymer solutions and then the porous membrane itself. Some of these membranes were further investigated as their surface and cross-sectional morphologies allowed us to delve into the membrane performance.

In this study the ternary phase diagrams were not determined, instead of this, the previous studies^32,50^ were taken into account to gain insight into the starting point of the polymer solution formulation. Jue *et al.*^32^ reported the preparation of the hollow fiber PIM-1 membrane using THF, DMAc and ethanol in the dope composition for the electrospinning. It was observed that the pore formation starts below a dense layer on top of the membrane cross-section. This fact might be attributed to the presence of THF: as THF has a low boiling point (64 °C), it evaporates rapidly when the polymer solution is exposed to the air, resulting in the formation of a dense film on top of the membrane. Taking this situation into account, this current study aimed to use the lowest possible amount of THF in the casting solution.^33^ Therefore, another solvent with a high boiling point was needed to be used together with THF to facilitate pore formation at the desired moment. At this point, the most common non-volatile solvent NMP is the best candidate due to its excellent miscibility with water. However, NMP can only dissolve PIM-1 at low concentrations to achieve one phase solution. In this study, PIM-1/NMP/THF solutions were prepared, and our observations showed that the optimum concentration to obtain one phase solution is PIM-1/NMP/THF:12.5/69.5/18 wt%, which is the case for PM-6 and PM-9. Higher NMP loading in the casting solution caused insufficient dissolution of PIM-1.

The characterization of PM-6, PM-9, PM-11 and PM-13 porous PIM-1 membranes were carried out by FTIR measurement. FTIR spectrum of each membranes showed the characteristic peaks corresponding to each functional group. FTIR spectrum of the membranes can be found in ESI, Fig. S1.†

### 3.2 Membrane morphology

**Fig. 1:**
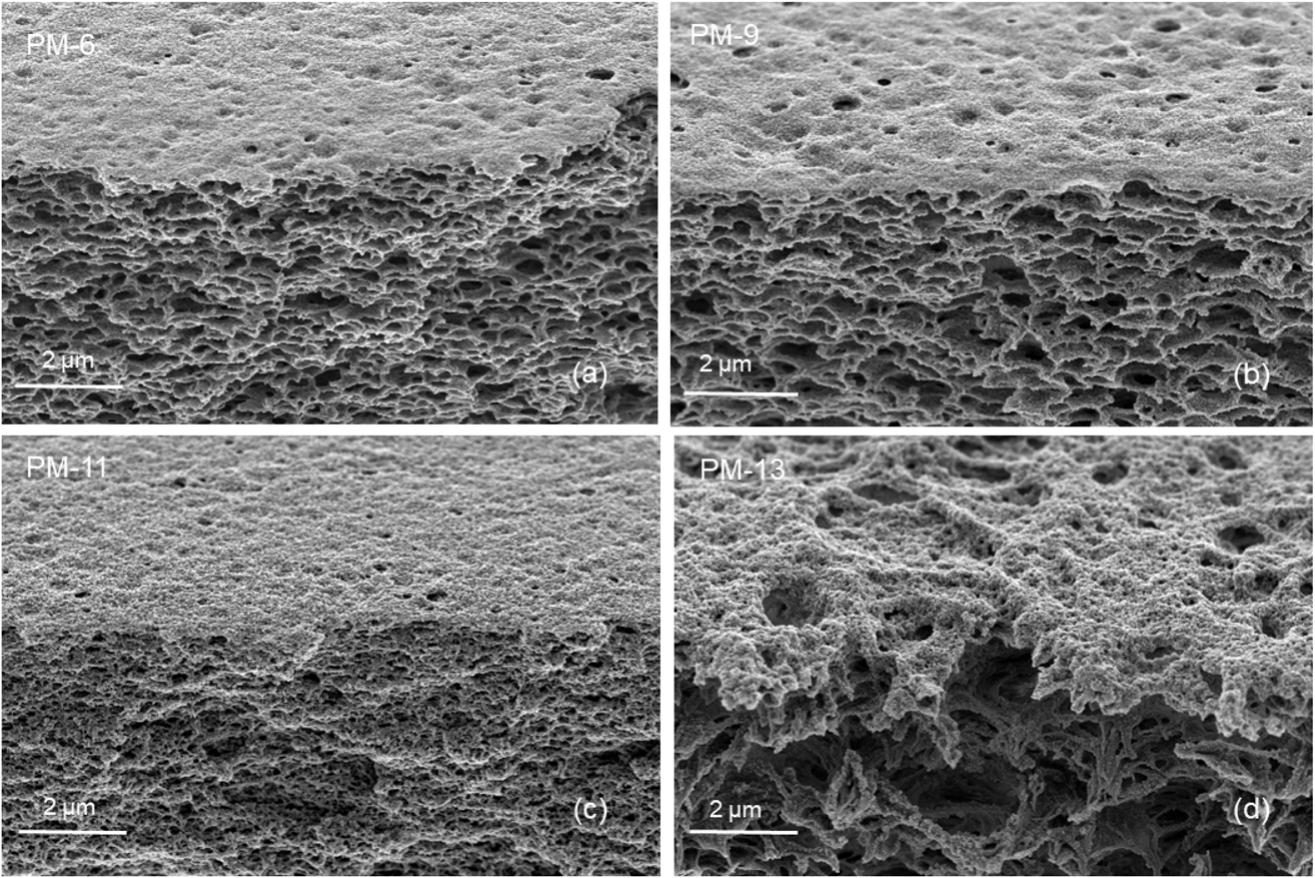
Cross-sectional images of PM-6 (a), PM-9 (b), PM-11 (c) and PM-13 (d) (2 μm).

The SEM images of PIM-1 membranes are shown in this section to demonstrate the internal and surface morphology of the membranes. Fig. 1 shows the cross-sectional morphology of PM-6, PM-9, PM-11 and PM-13 with well-developed internal porosity of the membranes. Fig. 1a and b show the presence of the interconnected pores within the membrane, which are covered by a denser but still visibly porous layer on top of the membrane. According to the generally accepted classification, microfiltration membranes have a pore size of 100–10000 nm, while ultrafiltration membranes have a pore size in the range of 2–100 nm.^39^ The membrane PM-11 shown in Fig. 1c has a much more compact morphology with smaller voids than PM-6 and PM-9. In case of PM-11 8 wt% THF resulted in very different morphology of the membrane in the vicinity to the surface exposed to the air during the membrane formation. One can speculate that a small THF content in the polymer solution led to smaller changes in polymer concentration when THF was unavoidably evaporated from the polymer solution surface, and this prevented the formation of a distinctive “crust” as on the surface of PM-6 and PM-9. Yong *et al.*^51^ pointed out that a higher polymer concentration in the casting solution suppresses the formation of macrovoids, resulting in the formation a compact porous structure. The formation of internal pores increases in PM-13 compared to PM-6, PM-9 and PM-11as shown in Fig. 1d. In this case the polymer concentration is slightly lower than in PM-6 and PM-9, but in addition the polymer used to cast PM-13 is from the different batch (PM-HT, see Table 1), which has a high molecular weight. PM-13 therefore clearly demonstrates the effect of molecular weight on the membrane formation process, where a higher molecular weight of the polymer leads to the formation of a very open porous structure both on the surface and within the membrane.

**Fig. 2:**
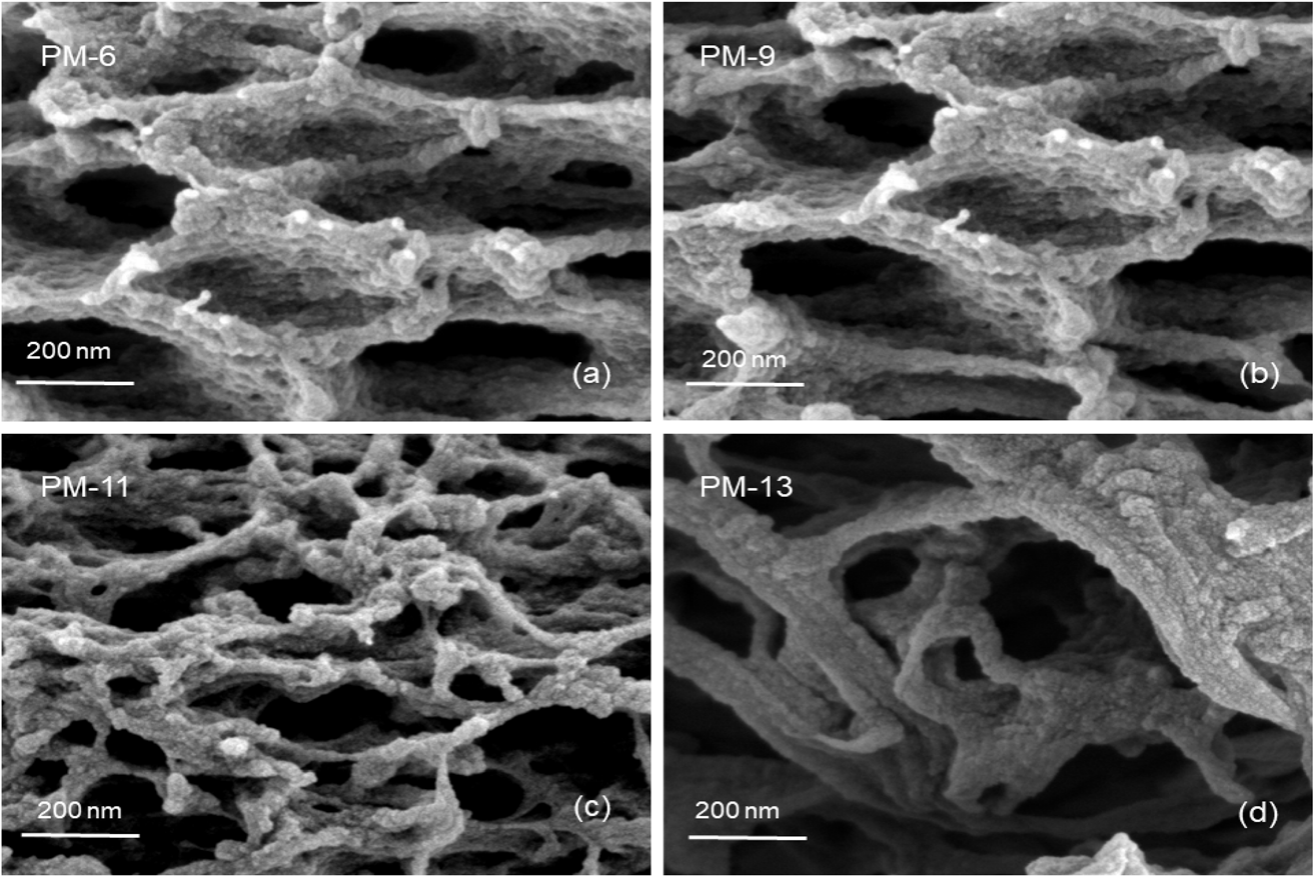
Cross-sectional images of PM-6 (a), PM-9 (b), PM-11 (c) and PM-13 (d) (scale bars: 200 nm).

Fig. 2 provides a magnified visualization of the cross-sectional morphology of the membranes. From Fig. 2a and b it can be said that PM-6 and PM-9 have similar pores both in shape and in size as one can expect for membranes formed of the same polymer and from the polymer solution of the same composition. On the other hand, PM-11 has smaller voids that are well interconnected (Fig. 2c). This difference in porous structure should result from the difference in polymer concentration between 12.5 wt% for PM-6 and PM-9, and 15 wt% for PM-11. PM-13 on Fig. 2d shows large pores with walls formed of a denser, fiber-like polymer. The difference of PM-13 to other membranes is high molecular weight of the polymer. Presumably, this parameter even at the lowest polymer concentration and the highest THF concentration in the casting solution, has led to changes in the interaction of PIM-HT with water during the phase inversion process, resulting in both a highly porous internal structure of the PM-13 and large pores on the membrane surface (Fig. 1d).

**Fig. 3:**
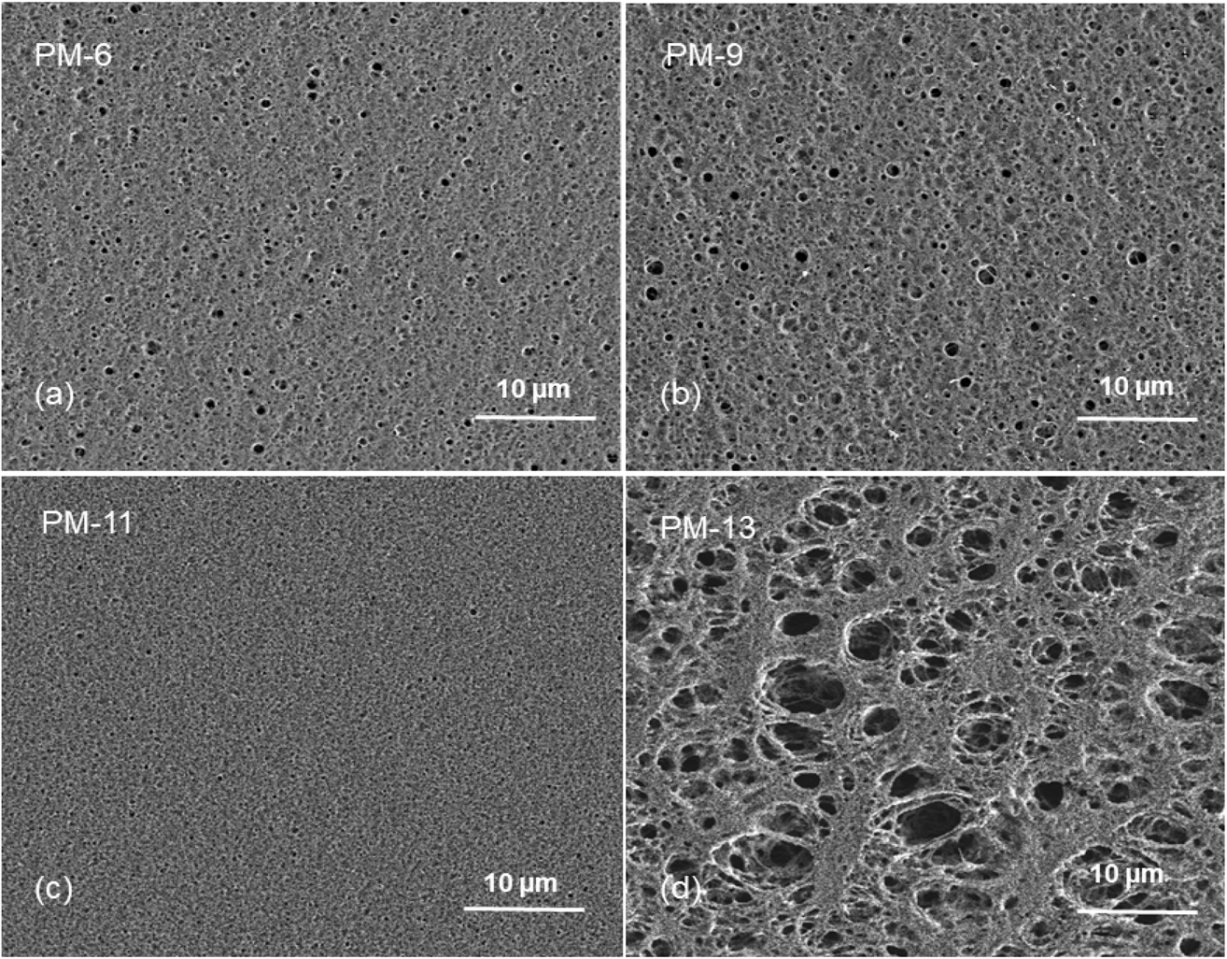
Surface morphology of PM-6 (a), PM-9 (b), PM-11 (c) and PM-13 (d) (scale bars: 10 μm).

**Fig. 4:**
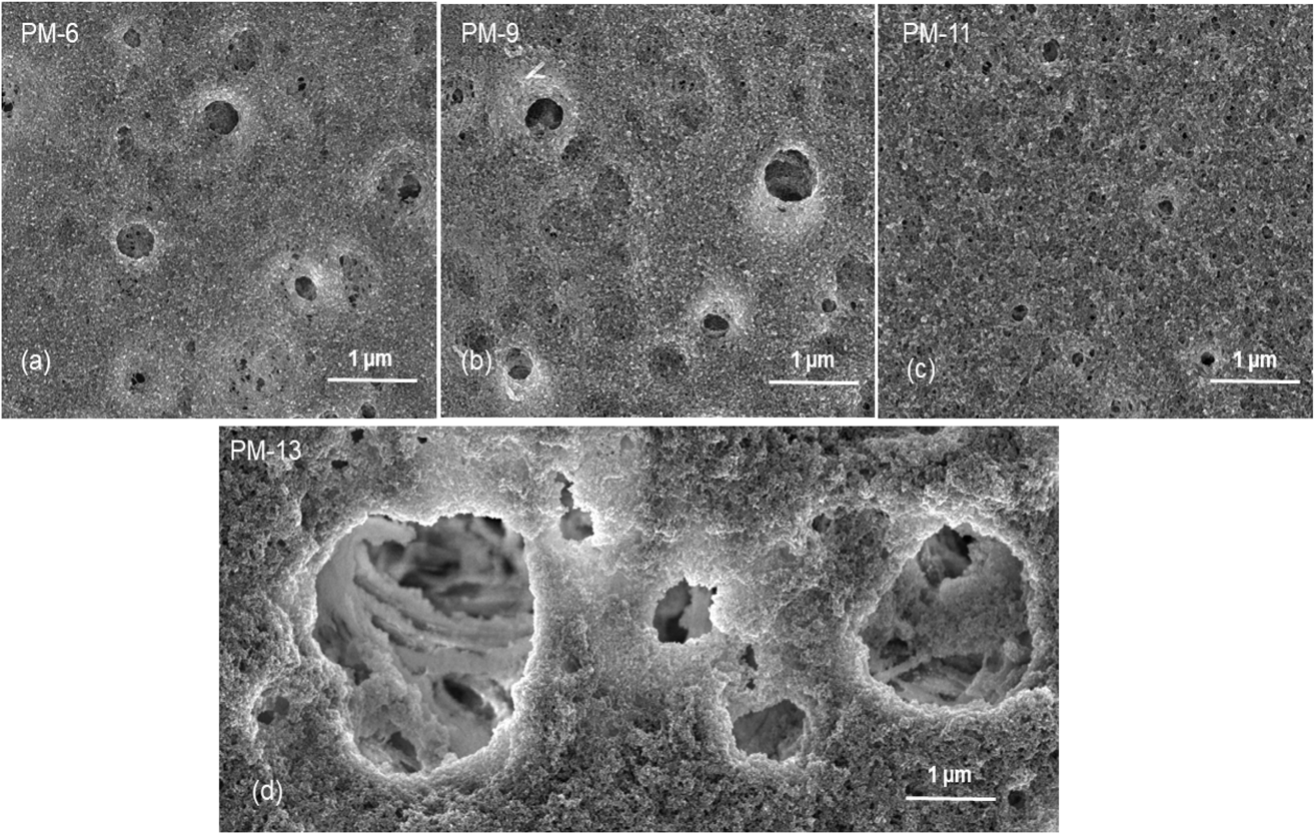
Surface morphology of PM-6 (a), PM-9 (b), PM-11 (c) and PM-13 (d) (scale bares: 1 μm).

Fig. 1 already showed that the cross-sectional morphology of the membranes is anisotropic, the feed side of the membranes is covered by a layer denser than the bulk of the membrane. For a better understanding of the surface porosity of the membrane, SEM images of the membrane surface are shown in Fig. 3. Fig. 4 shows SEM images of the membrane surface with enlarged magnification. Fig. 3a–c, corresponding to PM-6, PM-9 and PM-11 respectively, clearly demonstrate the presence of small pores of different sizes with the smallest visible pore size in case of PM-11, while PM-13 in Fig. 4d shows large surface pores through which the internal structure of the membrane can be observed. The major difference of PM-13 that distinguishes it from PM-6, PM-9 and PM-11 is the higher molecular weight of the PIM-HT (Table 1). It is a questionable whether the formation of big pores is due to the higher molecular weight of the polymer, as the membrane casting technique is the same for all membranes. The polymer content in the PM-13 case is the lowest of all the membranes and the THF content in the polymer solution is the highest. The high THF content should have led to an increase in the polymer concentration near the surface of the polymer solution exposed to the air due to the intensive evaporation of the solvent. At the same time, the decrease in THF concentration should lead to a lower polymer solubility in the NMP/THF system, since, as mentioned above, the PIM-HT has limited solubility, due to its high molecular weight. The combination of these factors with the highest weight factor of the polymer molecular weight resulted in the formation of the membrane with the largest surface pores and voids within the membrane. Prior studies have noticed the effect of the molecular weight on the structure and performance of ultrafiltration membranes.^52–55^ Zhou *et al.* showed that increasing PES molecular weight led to the formation of larger pores on the membrane surface.^53^ However, Miyano *et al.* asserted that the molecular weight is the least factor, which influences the pore size of the membrane while the concentration of polymer solution and solvent choice are more dominant parameters.^54^ Another remark on molecular weight influence on membrane morphology was made by Haponska *et al.*, who claimed that lower molecular weight caused a compact morphology of the membrane cross-section while higher molecular weight led to a more spongy-like porous structure.^55^ Taking these findings into consideration, it can be concluded that the molecular weight has a strong impact on the formation of the membrane structure, especially in case when the polymer is only partly soluble in the main solvent of the solvent system.

### 3.3 Water flux measurement

Experiments on the liquid water flow transport through porous membrane pursued two aims: determination of LEP and investigation of water transport at 4 bar pressure as driving force to be able to compare results with other developed microfiltration (MF) and ultrafiltration (UF) membranes. Unfortunately, the experimental LEP determination did not give conclusive results, at transmembrane pressure as low as 0.5 bar minor water flow was determined. It is in the line with the theoretically determined LEP values listed in Table 3. The transmembrane pressure for continuous water permeance measurements was chosen based on the pore size of the membranes used in this study. The average pore sizes calculated by SEM are between 0.1 and 0.2 μm as seen in Table 3. This range corresponds to the pore sizes of membranes used in microfiltration (10–0.1 μm) and ultrafiltration (0.1–0.002 μm).^56^ On the other hand, the transmembrane pressure can reach up to 4 bar in microfiltration and 2.5–5 bar in ultrafiltration.^57,58^ Considering that, the average pore size of the membranes in this study is in the MF and UF range, the transmembrane pressure was set to 4 bar to be in an application range. In addition, since there is a direct relationship between the applied transmembrane pressure and the permeate flux, this value was not set too low.^59^ Another reason for determining the transmembrane pressure is to prevent membrane compaction. In the case of transmembrane pressure between 2 and 70 bar, membrane compaction is considered negligible.^60^ All these reasons led to set the transmembrane pressure to 4 bar.

**Table 3:** Contact angle, pore size analysis and theoretical LEP of the porous membranes

	Contact angle (*θ*)	Average pore size (μm)	Largest pore (μm)	LEP (bar)
PM-6	106.9	0.14 ± 0.13	0.83	1.01
PM-9	113.4	0.21 ± 0.22	1.42	0.80
PM-11	97.9	0.11 ± 0.08	0.75	0.52
PM-13	129.6	—	5.36	0.34
PIM-1 (TFC membrane)	70 (in this study) 89 (ref. 61)	—	—	—

In addition to average pore size determination, Table 3 shows contact angle, largest pore size for each membrane and theoretical LEP values calculated from aforementioned parameters. It can be seen that the sequence of the largest pore size is consistent with the morphology images as PM-11 < PM-6 < PM-9 < PM-13. The theoretically calculated LEP follows the order of PM-13 < PM-11 < PM-9 < PM-6.

**Fig. 5:**
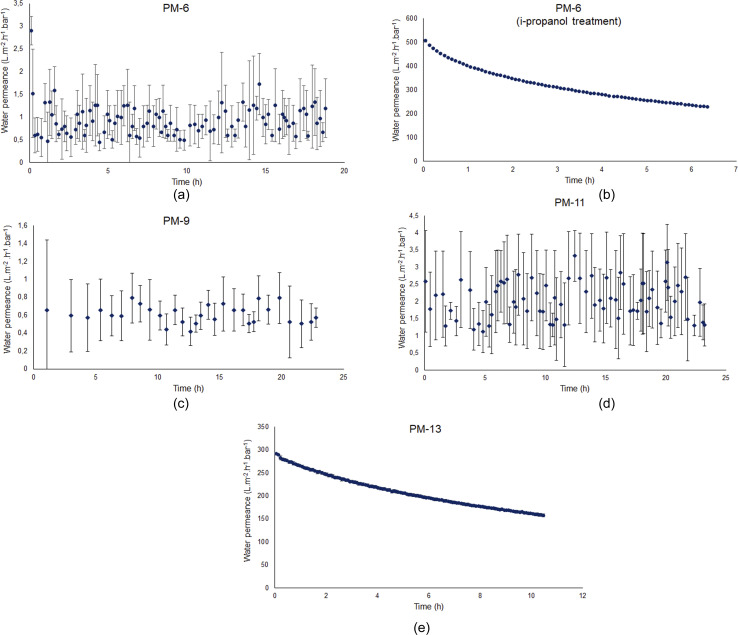
Water flux data of PM-6, PM-9, PM-11 and PM-13. Due to the resolution of the measuring range, it was difficult to record a constant flow. In order to show the deviations within a measurement error bars are shown.

Fig. 5 shows the time dependence of the water permeance for four membrane samples. These samples were chosen from a set of many developed in the current study (ESI, Table S1†). The decision to focus on PM-6, PM-9, PM-11 and PM-13 was taken based on the analysis of SEM images, which showed the developed porous structure both on the membrane surface and in the bulk of the membrane.

Water flux measurements were carried out using ultra-pure water. The applied transmembrane pressure of 4 bar is above the theoretical LEP for all samples and anyway water flow through PM-6, PM-9 and PM-11 is on the level of the measurement system resolution. The fact that water penetrates through the membrane shows that on the membrane surface pores are larger than those presented in the Table 3 and not identified by SEM investigation. The quantity of these pores is extremely small since no significant water flux can be observed under 4 bar pressure. Most of the membrane surface does not allow liquid water to pass through. In that point, membrane hydrophobicity or hydrophilicity should be considered since it significantly influences membrane performance. Contact angle of a dense PIM-1 film is about 90° (ref. 30 and 62) and measurements done for porous membranes under study revealed that the contact angle of porous PIM-1 membranes are higher (Table 3, 97–130°) than that of a dense film. The reason for this could be that the contact angle can vary depending on the surface morphology.^63,64^ For highly hydrophobic membranes, it is hard to evaluate the accessibility of the porous system for liquid water and its ability to transport water.

To understand the ability of the developed porous membranes to the transport of liquid water, one of the samples, PM-6, was immersed in the isopropanol–water mixture and soaked with the liquid. The alcohol–water mixture has a low surface tension and low contact angle toward PIM-1 and it allowed for effective impregnation of the pores with water miscible liquid.^65^ Several publications have shown that membranes treated with wetting agents such as ethanol or isopropanol demonstrate higher water flux even after alcohol evaporation.^66–68^ It should be noted that the pore activation by alcohols is a temporary effect since the wetting agent adsorbs only physically on the pore surface and do not cause any chemical modification. Nevertheless, it would be beneficial to investigate a PIM-1 membrane treated by a wetting agent in order to discuss its porosity. Therefore, we conducted an additional post-treatment for PM-6 which exhibits the smallest pore size according to SEM results. For this reason, PM-6 was soaked into an isopropanol–water mixture in order to make the membrane hydrophilic to investigate whether the pore openings occur. Fig. 5b depicts that the water flux of PM-6 significantly escalates to 500 L m^−2^ h^−1^ bar^−1^ after the membrane were exposed to isopropanol–water treatment, which triggered the interconnection of the small pores. It might be explained by the fact that small pores do not take part in filtration at the beginning, since the surface tension between liquid–solid is less than that of liquid-air. After the wetting treatment, cohesive forces between water molecules becomes weaker, therefore, water can penetrate into pores.^68^ If we want to compare this result with a reference polymeric membrane, a polyethersulfone (PES) membrane can be considered. This polymer membrane is widely used in ultrafiltration and microfiltration and a vast amount of studies on PES membranes can be found in the literature. Studies have shown that PES membranes exhibit water flux in the range of 300–500 L m^−2^ h^−1^ bar^−1^.^69,70^ Therefore, PM-6 can show a water flux at the level of PES membranes, by virtue of pore activation after isopropanol–water treatment. If it is to be compared with polymers used in membrane distillation, the example of PVDF and PTFE can be given. Nawi *et al.*^71^ recorded the pure water flux value of pristine PVDF membrane as approximately 200 L, while Yu *et al.*,^72^ in their work on PTFE for MD applications, reported a pure water flux value of approximately 900 L. Based on these reference values, it can be stated that the PM-6 membrane after isopropanol treatment exhibited a notable water flux of 500 L m^−2^ h^−1^ bar^−1^. According to this result, it can be inferred that PM-6 acquires pores but relatively in small size which hinders the liquid water transport through the virgin membrane. It should be noted that since the polymer was casted on a porous nonwoven support, the actual thickness of the polymer cannot be determined in this study by excluding the nonwoven support. Therefore, we are limited to determine of the membrane porosity. Yet, it allows us to interpret the effect of isopropanol–water treatment on the pore activation and water flux.

A similar water flux result was obtained from PM-9 as expected since it is the same PIM-1 which was used in PM-6, yet PM-9 was obtained from another membrane casting in order to reproduce the results. Fig. 5c exhibits the absence of water flux. As mentioned before, water molecules are not able to transfer through the membrane pores at 4 bar transmembrane pressure in this study. Likewise, PM-11 demonstrates also scattered but extremely low water flux values as seen in Fig. 5d, what cannot be considered as water flux. This finding is anticipated due to the surface morphology and internal porosity of PM-11 are not appropriate to transfer the water molecules across the membrane. Experimentally, although the transmembrane pressure is actually above the theoretically calculated LEP for all membranes, PM-6, PM-9, and PM-11 showed basically no water flux (only noise). This can be attributed to the deviation of the experimental LEP from its theoretical value. Despite operating the water flux experiments with a transmembrane pressure higher than the theoretical LEP, the reason for the water flux remaining only as noise could be the variations in the actual values according to the operating conditions. For example, the presence of defects in pore structure or the pore shape not being uniformly cylindrical as assumed in reality can cause deviations between calculated values based on theory and experimental results.^73,74^ However, the observed difference in water flux measurements is associated to PM-13. Unlike the other membranes studied in this work, Fig. 5e shows that PM-13 provides a significantly high water flux of 300 L m^−2^ h^−1^ bar^−1^. This result might be attributed to the large pores of PM-13 on the surface and cross-section of the membrane and therefore PM-13 possesses the lowest LEP value. It can therefore be assumed that 4 bar transmembrane pressure is enough for PM-13 to press the water molecules through the membrane pores without the necessity of a wetting treatment. Isopropanol–water treatment was not applied to all membranes. Since PM-13, which has the largest pore according to SEM results, already showed some water flux, it was concluded that the membrane with the smallest pore could be used to see if such a treatment would have an effect, and therefore only PM-6 was exposed to isopropanol soaking.

### 3.4 Water vapor permeance

MD is the method of water purification by transport of water molecules as vapor through the porous structure of the membrane. The ability of the developed membranes to transport water in a vapor form was studied on an in-house designed “pressure increase” facility. On this facility it is possible to study transport of gases and vapor though flat and hollow fiber shaped membranes in relation to temperature in the range of 4–120 °C and feed pressure in the range of 20–1200 mbar. For water vapor, the maximum achievable feed pressure applied to the membrane was *ca.* 95% of vapor activity at the temperature of experiment. Permeate pressure range in which permeance data points are acquired can be chosen in accordance with membrane performance. For slow membranes it can be 0.1–0.2 mbar abs, for “fast” membranes 1–13 mbar abs. From the MD point of view, the obvious drawback of this facility is the inability to start an experiment at saturated vapor pressure. At the same time, the feed vapor activity can be as high as 95%, very close to the saturation pressure and at the same time the membrane can not be exposed to liquid water due to *e.g.* condensation caused by a temperature drop on the membrane surface as a result of the Joule-Thomson effect.^75^

**Fig. 6:**
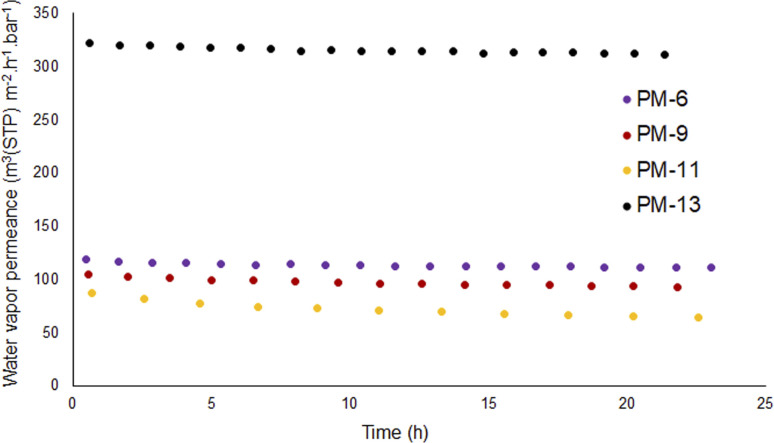
Water vapor permeance of PM-6, PM-9, PM-11 and PM-13.

Each membrane was exposed several times to the same pressure of vapor; during each exposure, several points were collected to have statistically relevant data and the experiment was repeated after the membrane was fully evacuated. Fig. 6 shows that all membranes have significant permeance for water in a vapor form while Fig. 5 showed that membranes PM-6, PM-9 and PM-11 had extremely limited transport when liquid water was applied under a transmembrane pressure of 4 bar.

Fig. 6 shows that PM-6, PM-9 and PM-11 show high water vapor permeance with the highest value for PM-6. This difference can be considered insignificant since these are handmade casted membranes and THF evaporation is not controlled. On the other hand, PM-13 exhibits dramatically higher water vapor permeance which is almost 3-fold of PM-6. This result can be attributed to large surface pores of PM-13 and well interconnected inner porosity which allows water transport without any obstruction. To compare water transport in vapor and liquid forms, vapor permeance was converted to a comparable unit as shown in Table 4. It is seen that the vapor/liquid permeance ratio is tremendous. Especially in case of PM-6 and PM-9 which have the smallest pores, this comparison vividly demonstrates how water permeance differs in the vapor and liquid phases. In particular, our findings up to this point are supportive of the use of porous PIM-1 membranes in MD considering that this process requires a hydrophobic membrane which would allow only vapor to permeate. As it can be seen from Table 4 measures to prevent membrane wetting are essential since as soon as the membrane is impregnated with water the flux of liquid contaminated liquid will be significantly higher than that of vapor.

**Table 4:** Comparison of water transport through PIM-1 membrane when membrane is exposed to vapor or liquid on the feed side

Sample	Vapor permeance (m^3^(STP) m^−2^ h^−1^ bar^−1^) (Fig. 6)	Vapor permeance (kg m^−2^ h^−1^ bar^−1^) (Fig. 6)	Liquid flux (L m^2^ h^−1^ bar^−1^) (Fig. 5)	Permeance ratio
PM-6	125	100	1.5	67
PM-6^a^	125	100	500	0.2
PM-9	100	80	0.65	124
PM-11	85	68	2.0	34
PM-13	320	257	300	0.86

a PM-6 data from Fig. 5b when membrane was impregnated with water miscible fluid and exchanged to water during water flow experiment.

**Fig. 7:**
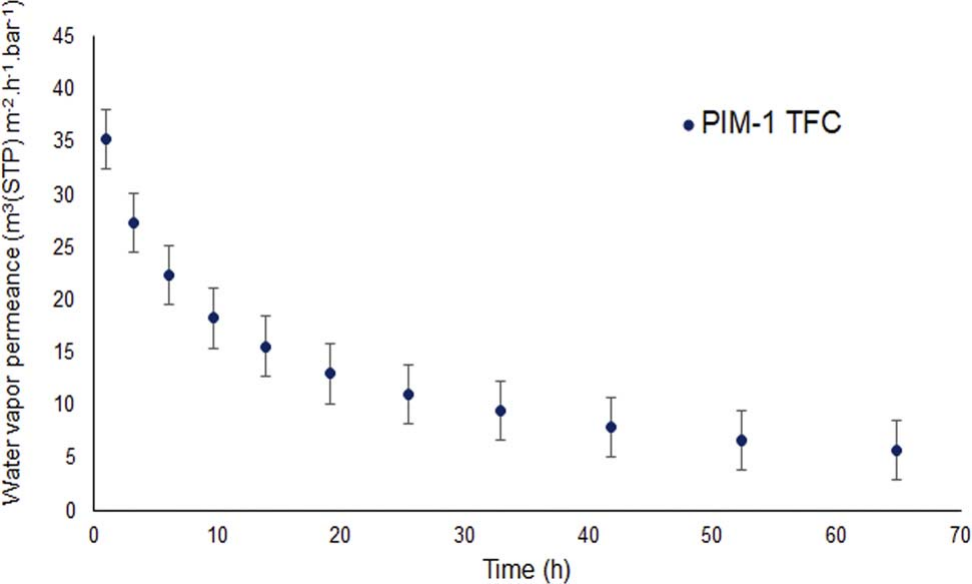
Water vapor permeance of the PIM-1 thin film composite (TFC) membrane.

To better understand the water vapor characteristics of porous PIM-1 membranes, it is beneficial to make a comparison with water vapor properties of a PIM-1 thin film composite (TFC) membrane. Fig. 7 depicts the water vapor permeance of PIM-1 TFC membrane determined at 40 °C. It should be noted that water permeance could not be measured at saturated vapor pressure due to the facility conditions. The water permeance shown in Fig. 7 was obtained at 91% of vapor activity. The permeance starts around 30 m^3^(STP) m^−2^ h^−1^ bar^−1^ and decreases almost 60% over time of 25 h to 10 m^3^(STP) m^−2^ h^−1^ bar^−1^. This drop in permeance reveals physical aging of the PIM-1 selective layer. However, it is observed that porous PIM-1 membranes sustain the water vapor permeance over time unlike the PIM-1 TFC membrane. Moreover, porous membranes demonstrate significantly higher water vapor permeance than the PIM-1 TFC membrane. However, in case of extreme water purity is required, a dense PIM-1 membrane might be better candidate.

In this study, the porosity of the membranes was not investigated by an analytical method. Nevertheless, prior studies have shown that Knudsen diffusion is an indication of membrane porosity. Basically gas transport in membranes might take place in different ways: poiseuille diffusion (wide pore size), Knudsen diffusion (intermediate pore size), molecular sieving and surface diffusion (small pore size) and solution-diffusion (dense membrane).^76^ In some cases, two or more of these diffusion mechanisms can occur concurrently. Among them, Knudsen diffusion occurs if the mean free path of the gas molecule is larger than the pore size of the membrane. Moreover, gas transport in Knudsen diffusion occurs in the gaseous state without participation of adsorption.^77^ Knudsen permeance is expressed as in eqn (3) where *ε* is the porosity of the membrane, *d*_p_ is the pore diameter, *τ* is the tortuosity, *L* is the thickness of the membrane, *R* is gas constant, *M* is molecular weight and *T* is the operating temperature.3
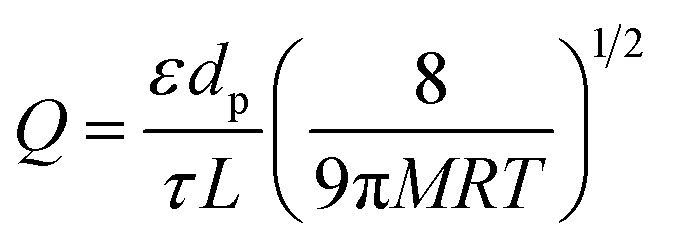


**Fig. 8:**
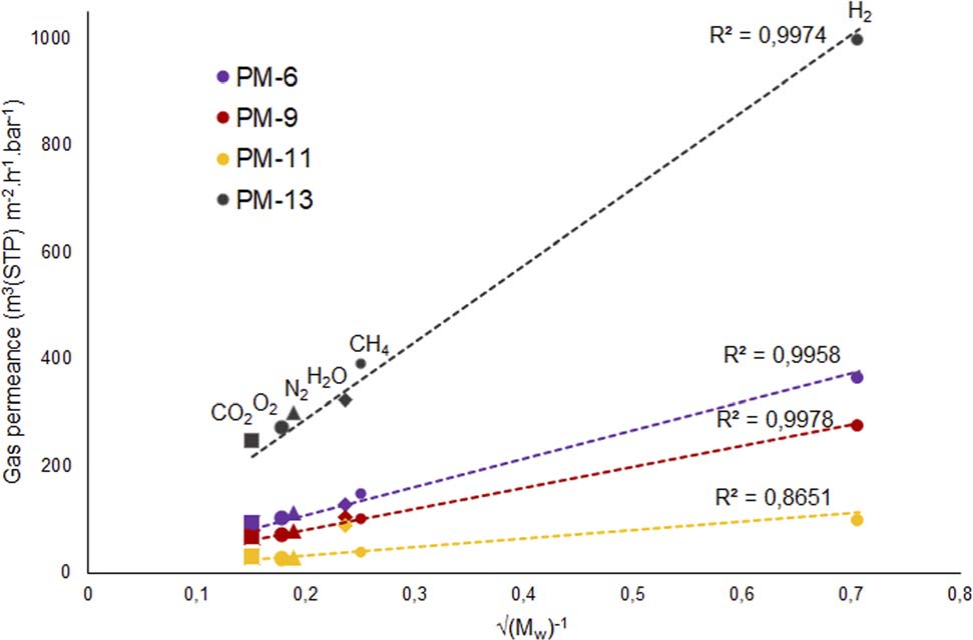
Knudsen diffusion dependency of PM-6, PM-9, PM-11 and PM-13.

According to this equation, Knudsen permeance is proportional to the inverse square root of both the molecular weight and the temperature of the permeate gas. From this point of view, the permeance values of the gases were plotted against the inverse square root of their molecular weight in order to discuss the porosity of the subjected membranes in this study. Fig. 8 shows a strong relationship between the permeance and the square root of the molecular weight of the gas molecules for PM-6, PM-9 and PM-13 which correspond with Knudsen diffusion. It is seen that the permeance of each gas follows in the order of the square root of molecular weight. Further to that, the curves show good regression fits (*R*^2^ = 0.992–0.9882) which is also attributed to the presence of significant Knudsen diffusion where molecule-pore wall interaction is dominates molecule–molecule interactions.^78^ It is important to bear in mind that Knudsen diffusion describes the gas transport through the pores. Therefore, it is limited by the lack of information on surface porosity. From our results it is unfortunately hard to evaluate if a combination of different diffusion mechanisms also exists in the membrane. Nevertheless, it should be noted that both Knudsen and Poiseuille diffusion might take part simultaneously in one membrane.^79^ Among the porous membranes studied in this paper, PM-11 is the exceptional case in terms of Knudsen diffusion. Water vapor permeance does not fit to the Knudsen plot as seen in Fig. 8. This might result from a combination of more than one diffusion mechanism in one membrane.

**Fig. 9:**
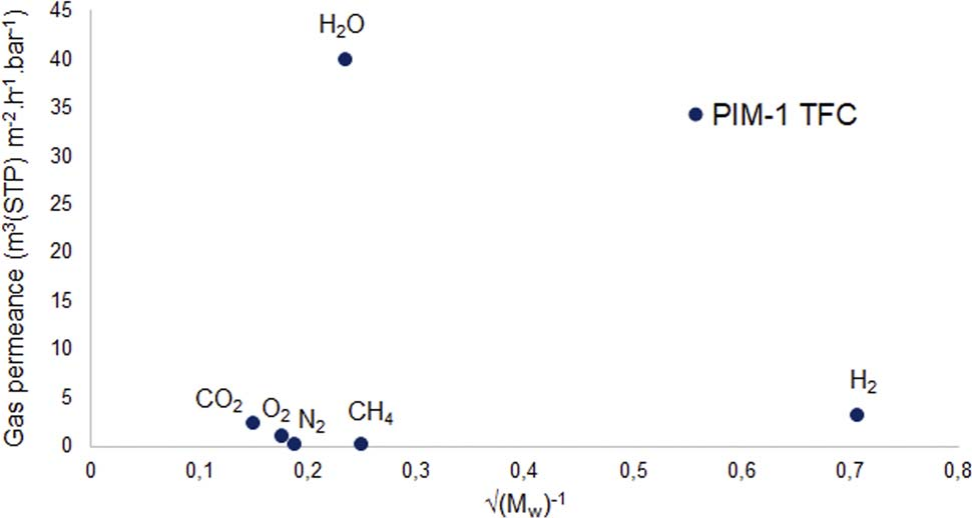
Knudsen diffusion dependency of the PIM-1 TFC membrane.

On the other hand, Fig. 9 shows a different behavior of the PIM-1 TFC membrane. It can be clearly seen that the gas permeance of PIM-1 TFC membrane does not increase in proportion to the square root of the molecular weight of the gases. This can be attributed to the behavior of the membrane with a selective dense layer which is most likely correspondent to solution-diffusion mechanism.^80^

## 4. Conclusion

This study aimed at a detailed examination of porous PIM-1 membrane formation by non-solvent induced phase separation. Polymer solutions with a variety of solvent and non-solvent combinations were prepared in order to obtain continuous pores from the surface through the cross-section of the membrane. It is difficult to prepare a PIM-1 polymer solution because of the lack of volatile and water-miscible solvents with a high boiling point that can be used in the phase separation method. To address this, different solvent-non-solvent mixtures including DMAc, EtOH, DCB, NMP and THF were prepared in this study in order to prepare a proper polymer solution. Among the polymer solutions, an NMP/THF combination was found to be the best candidate to cast the membrane from a homogeneous PIM-1 solution, *i.e.* without the formation of a precipitate. Four different PIM-1 membranes (PM-6, PM-9, PM-11 and PM-13) were obtained by NIPS method by using different ratios of the components in the NMP/THF/PIM-1 combination. In order to observe the effect of molecular weight on pore formation, PIM-1 used in PM-6, PM-9 and PM-11 has a low molecular weight, while PIM-1 used in PM-13 has a high molecular weight. Moreover, SEM imaging was performed to examine the morphology of the prepared membranes. Among the membranes studied in this work, PM-6 and PM-9 showed favorable void formation on the surface and cross-section of the membrane. PM-13 exhibits large pores, which was attributed using PIM-1 with high molecular weight. Additionally, water contact angle measurements were performed on membrane surfaces to investigate membrane wetting by LEP correlation. According to that, the distinct patterns in the sequence of the largest pore size and theoretical LEP values were observed consistently across the membranes. Water transport was also discussed in order to understand the membrane performance. Water flux of PM-6, PM-9 and PM-11 were neglectable, while PM-13 showed high water flux which is consistent with the presence of large voids. On the other hand, PM-6 and PM-9 demonstrated higher water vapor permeance compares to PIM-1 TFC membrane which is considered as a dense membrane. It is interesting that PM-6 and PM-9 allow to water vapor pass through the membrane however, liquid water cannot be transferred across the membrane. This membrane configuration might be a good candidate for membrane distillation since it uses hydrophobic membrane which only water vapor can pass through the membrane. These observations help us to raise intriguing questions regarding the applicability of PIM-1 on water separation. For future work, it would be interesting to establish pressure breakthrough measurement in order to deeper investigate the water flux data of PIM-1 membrane. Further experimentation regarding the role of porous PIM-1 membrane would be worthwhile since PIM-1 is easy to handle and favorable polymer which can be promising for water separation applications.

## Conflicts of interest

There are no conflicts to declare.

## Supplementary Material

RA-014-D3RA08398E-s001
