# Supplementary material for: Pioneering the preparation of porous PIM-1 membranes for enhanced water vapor flow
Source: RSC Adv. 2024 Mar 22;14(14):9631–45. doi: 10.1039/d3ra08398e (PMC10958458; doi:10.1039/d3ra08398e)
Supplement: RA-014-D3RA08398E-s001 [file RA-014-D3RA08398E-s001.pdf]

Electronic Supplementary Material (ESI) for RSC Advances.
This journal is © The Royal Society of Chemistry 2024

Pioneering the preparation of porous PIM-1 membranes for enhanced water vapor flow

Esra Caliskan[1], Sergey Shishatskiy[1], Volker Abetz[1,2] ,Volkan Filiz[1*]

[1] Institute of Membrane Research, Helmholtz-Zentrum Hereon, Max-Planck-Str. 1, 21502 Geesthacht, Germany;

[2] Institute of Physical Chemistry, University of Hamburg, Martin-Luther-King-Platz 6, 20146 Hamburg, Germany

[*] Correspondence: volkan.filiz@hereon.de; Tel.: +49-41-5287-242

Supplementary Information

Table S1: Composition of casting solutions and membrane casting parameters

Name	Composition (%wt.)	Precipitation bath	Batch	Membrane casting thickness	M_w (kDa)
PM-1	PIM/THF/DMAc/EtOH: 17.5/67.5/12.75/2.25	Water	PIM-LT-1	200 µm	76
PM-2	PIM/THF/DMAc: 17.25/69.5/13.25	Water	PIM-LT-1	150 µm	76
PM-3	PIM/NMP/THF: 12.5/69.5/18	Water	PIM-LT-2	150 µm (on glass substrate)	76
PM-4	PIM/DCB: 10/90	MeOH	PIM-LT-2	150 µm (on glass substrate)	76

PM-5	PIM/DCB: 10/90	MeOH/BuOH 50:50	PIM-LT-2	150 µm (on glass substrate)	76
PM-7	PIM/NMP/THF: 13/80/7	MeOH	PIM-LT-2	150 µm (on glass substrate)	76
PM-8	PIM/NMP/THF: 14.5/80.5/5	Water	PIM-LT-2	150 µm	76
PM-10	PIM/NMP/THF: 13/79/8	Water	PIM-LT-1	150 µm	76
PM-12	PIM/NMP/THF: 11.5/72/16.5	Water	PIM-HT	150 µm	142
PM-14	PIM/NMP/THF: 13/80/7	Water	PIM-LT-2	150 µm	76

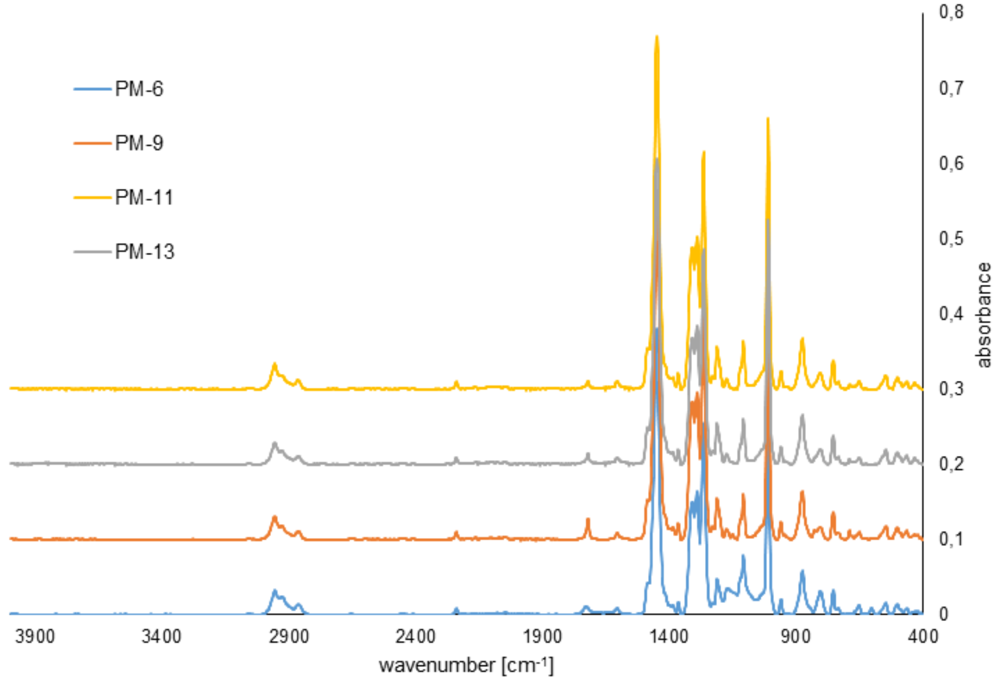

Figure S1: Comparative FTIR spectrum of PM-6, PM-9, PM-11 and PM-13

FTIR analysis was carried out to characterize the porous PIM-1 membranes which are PM-6, PM-9, PM-11 and PM-13. FTIR spectrum show the absorption bands at 2239 cm^{-1} and 1265 cm^{-1} which correspond to nitrile groups (C≡N streching). The absorption band between 2800-2900 cm^{-1} and at 1146 cm^{-1} are associated with CH_2 streching and bending vibration modes. The spectrum in the region at 1350-1250 cm^{-1} originates from C-O streching mode. Characteristic C-N streching appears at 1009 cm^{-1} and aromatic sp^2 C-H bending is visible at 874 cm^{-1}.

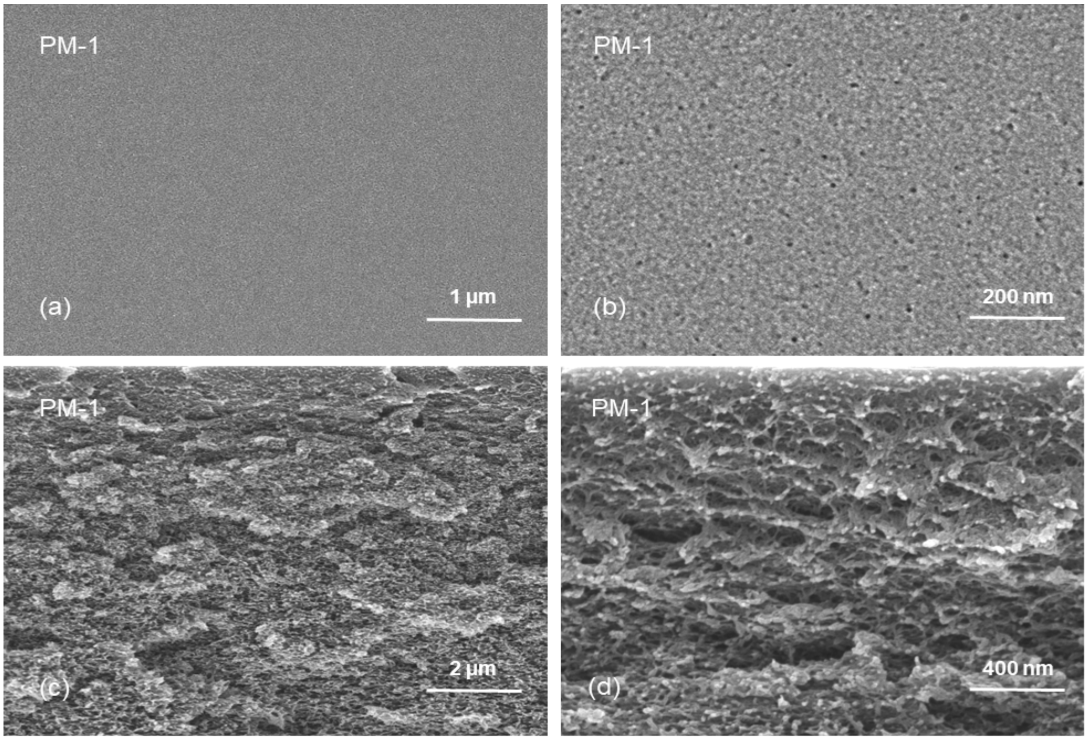

Figure S2: Surface (a, b) and cross-sectional (c, d) morphology of PM-1

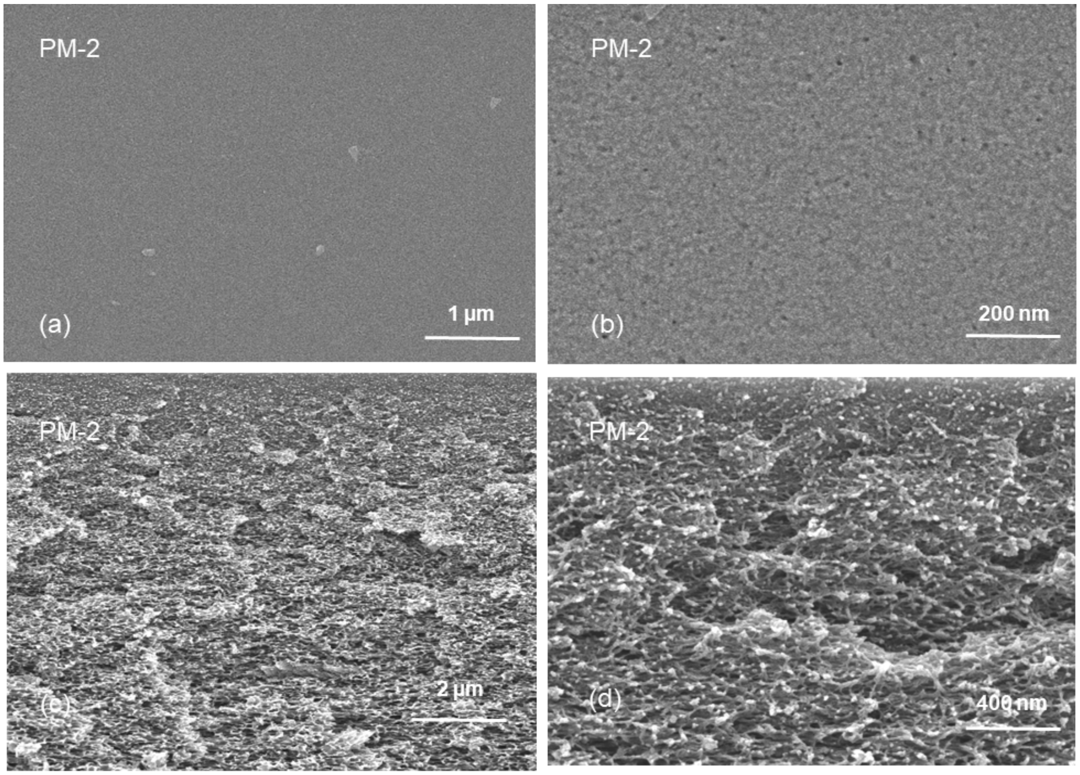

Figure S3: Surface (a, b) and cross-sectional (c, d) morphology of PM-2

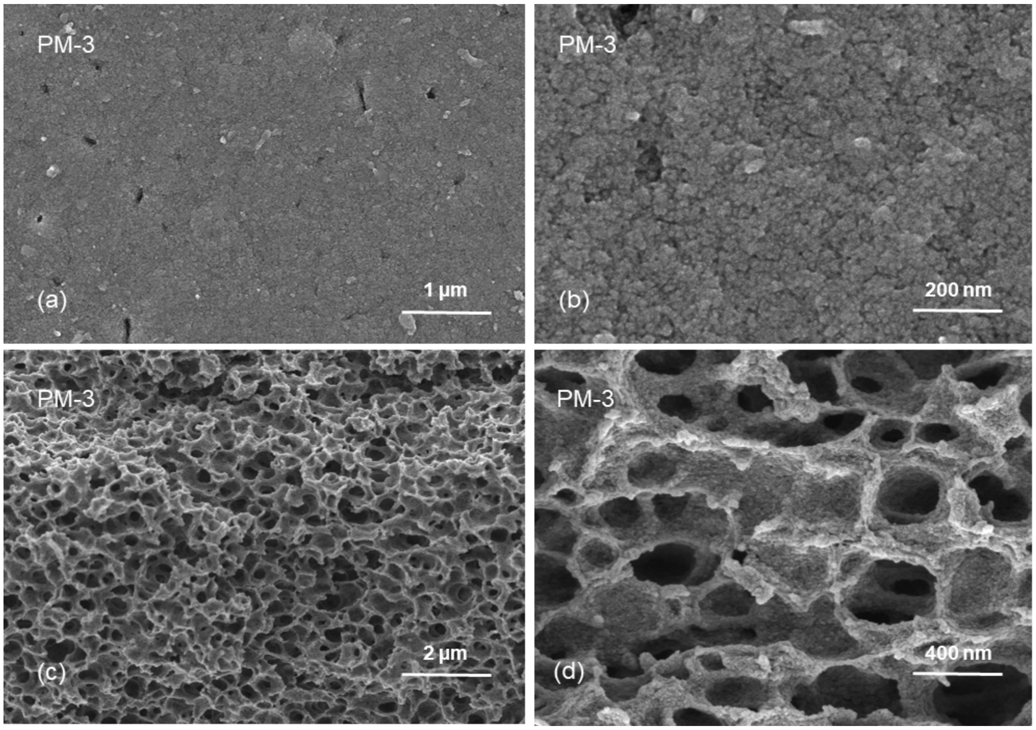

Figure S4: Surface (a, b) and cross-sectional (c, d) morphology of PM-3

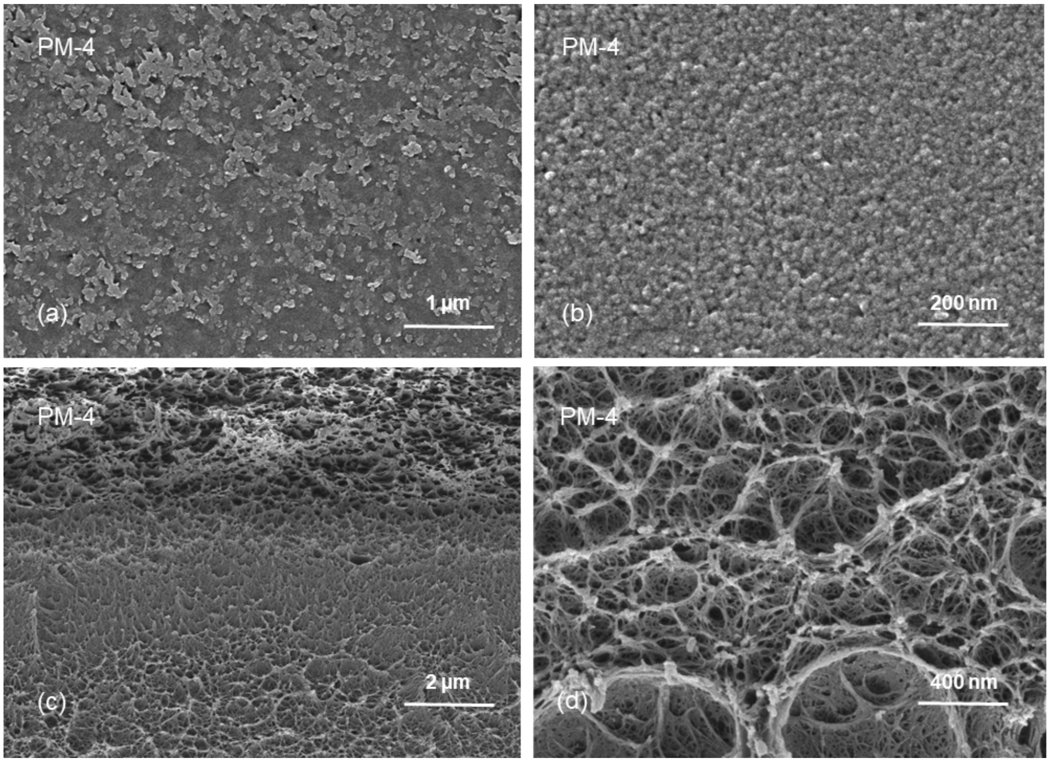

Figure S5: Surface (a, b) and cross-sectional (c, d) morphology of PM-4

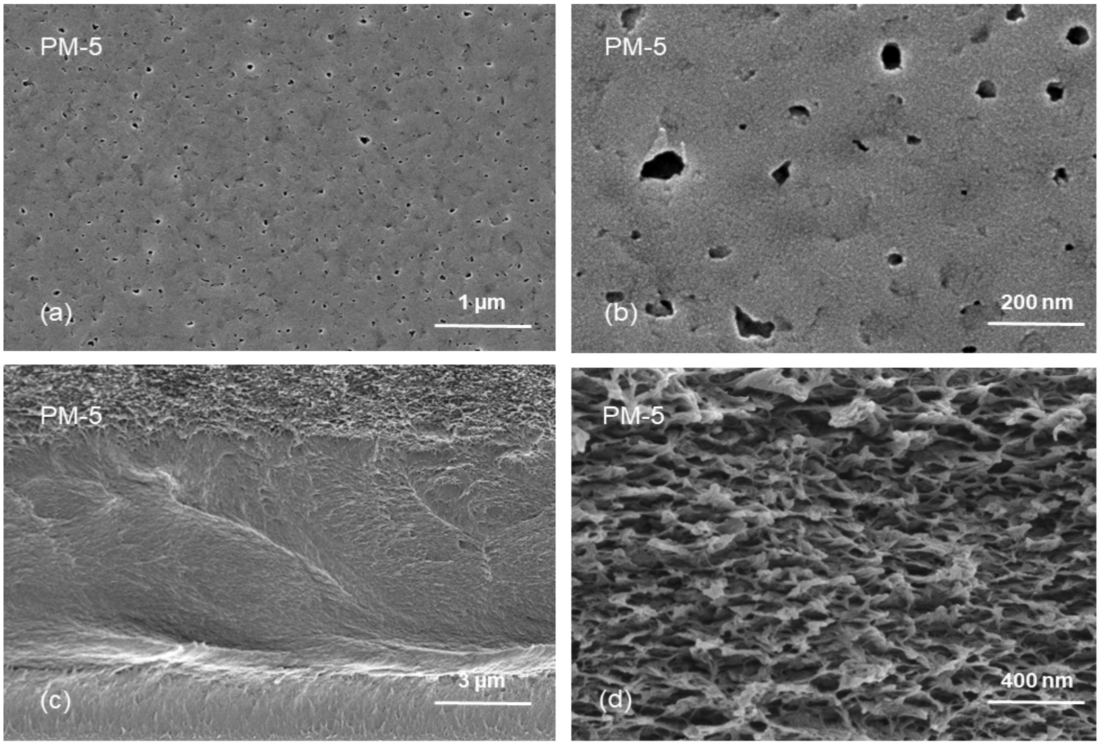

Figure S6: Surface (a, b) and cross-sectional (c, d) morphology of PM-5

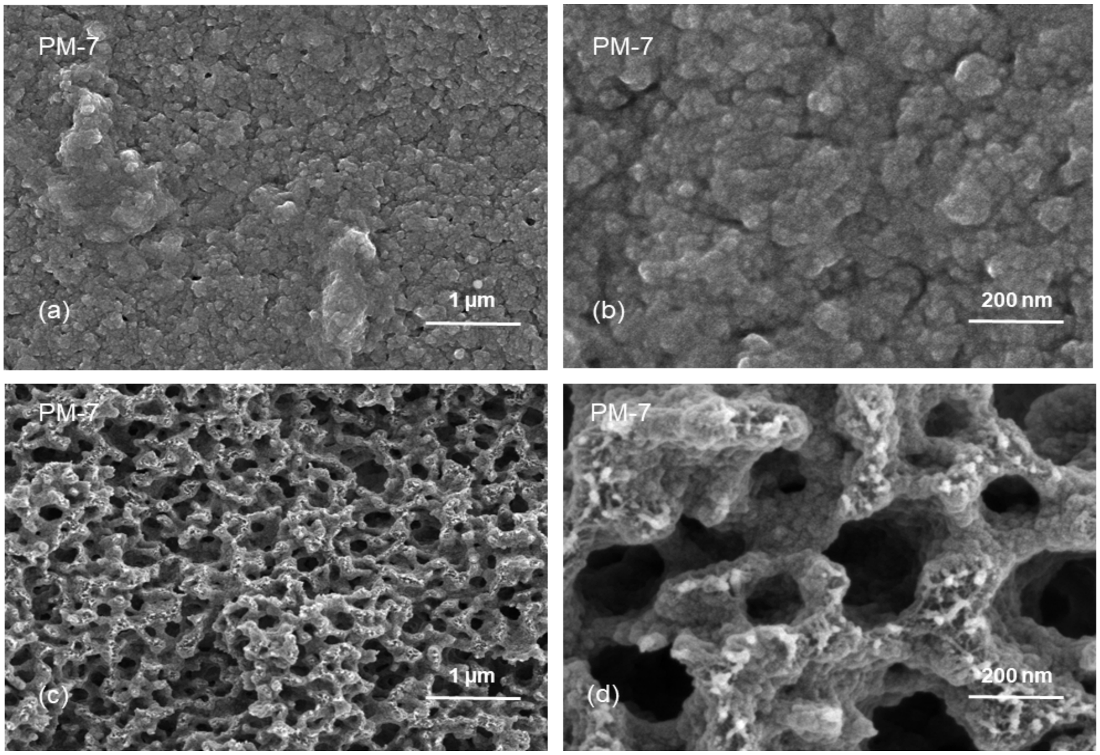

Figure S7: Surface (a, b) and cross-sectional (c, d) morphology of PM-7

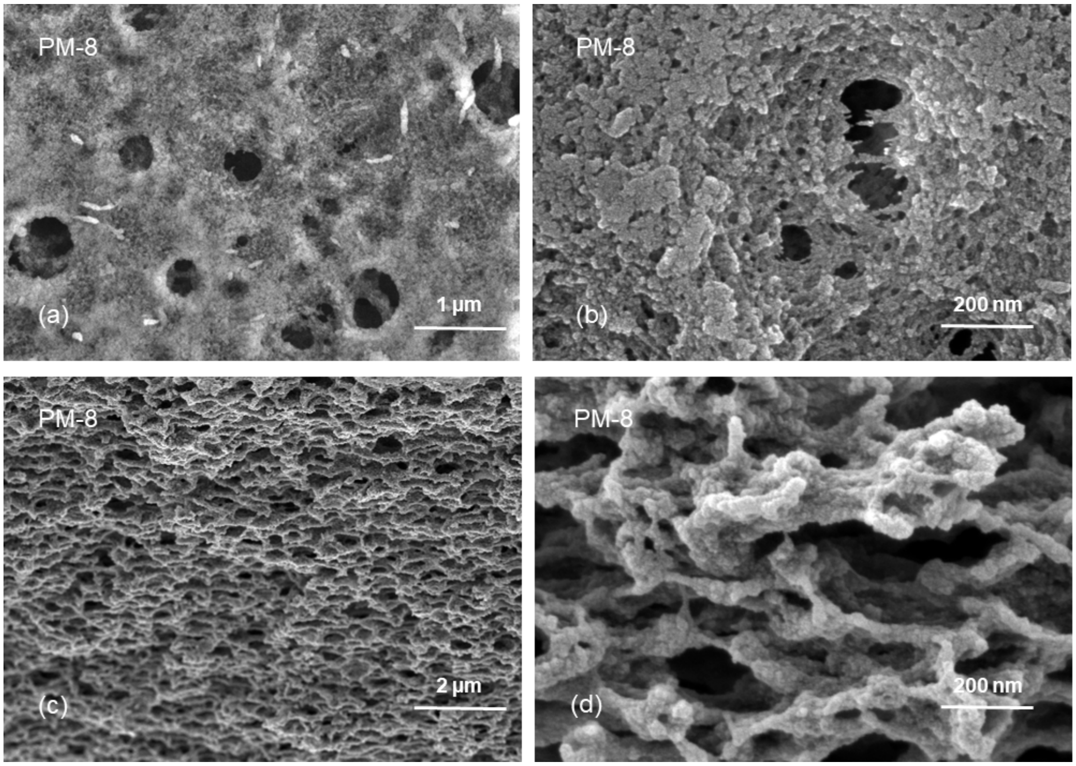

Figure S8: Surface (a, b) and cross-sectional (c, d) morphology of PM-8

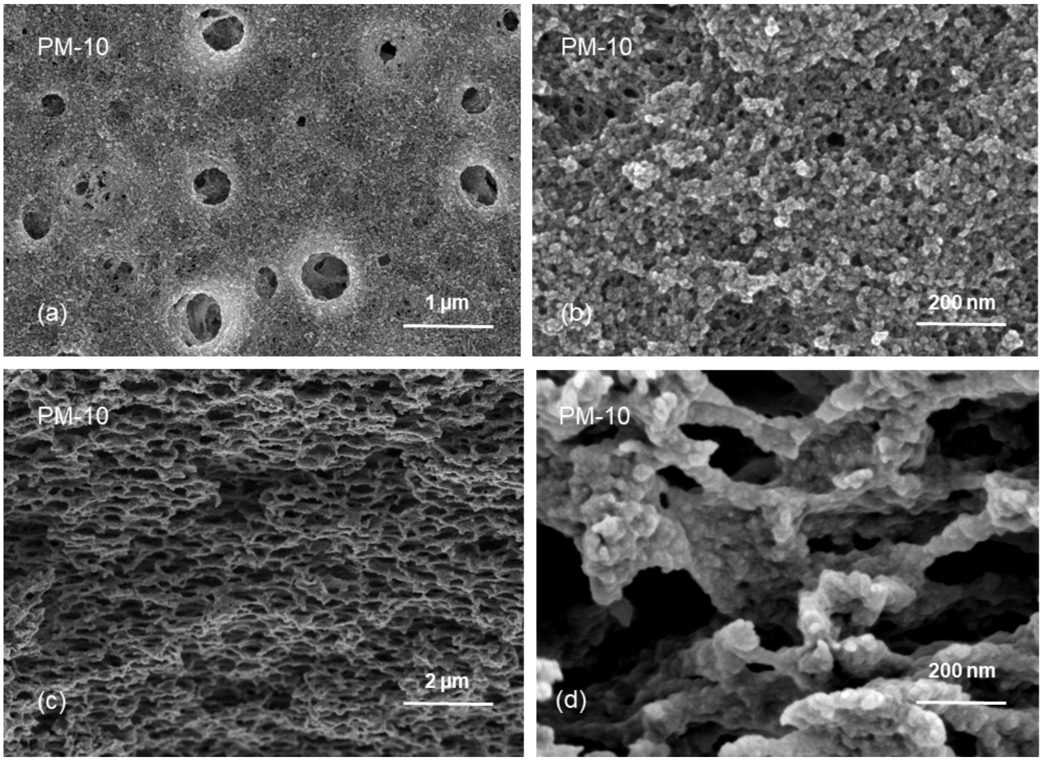

Figure S9: Surface (a, b) and cross-sectional (c, d) morphology of PM-10

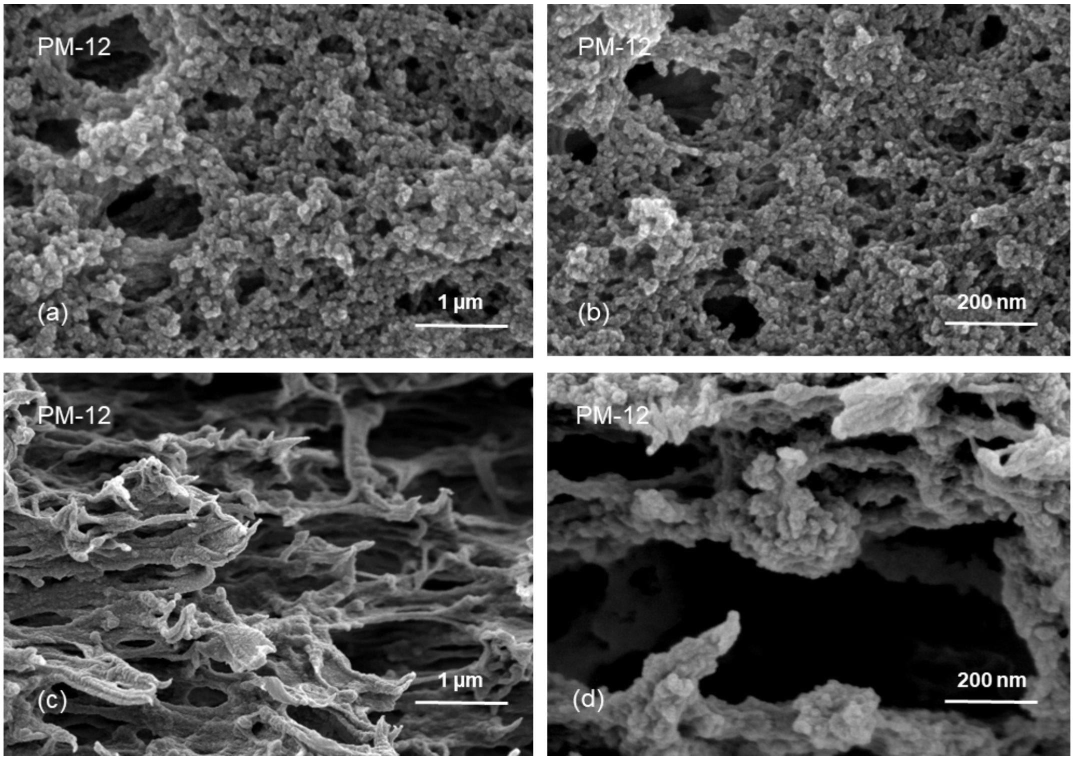

Figure S10: Surface (a, b) and cross-sectional (c, d) morphology of PM-12

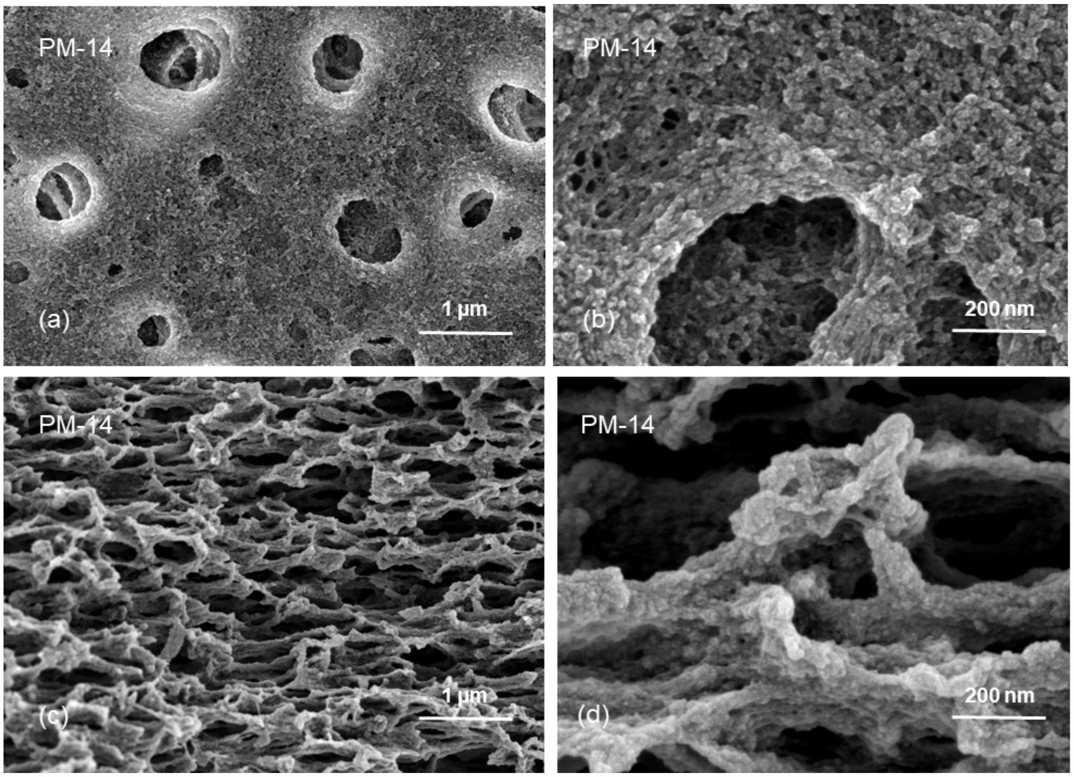

Figure S11: Surface (a, b) and cross-sectional (c, d) morphology of PM-14

Table S2: Water vapor permeance of PIM-1 membranes

	Water permeance ($m^3(STP)m^{-2}h^{-1}bar^{-1}$)	Batch
**PM-1**	35	PIM-LT-1
**PM-2**	42	PIM-LT-1
**PM-8**	124	PIM-LT-2
**PM-10**	350	PIM-LT-1
**PM-14**	201	PIM-LT-2

The measurement was performed at 30 ^{o}C and first measurement points of each membrane are displayed in **Table-S2**. PM-3, PM-4, PM-5 and PM-7 membranes were casted on a glass substrate. Therefore, water vapor permeance measurement could not be carried out since the membranes were not mechanically stable, it was difficult to handle these samples.

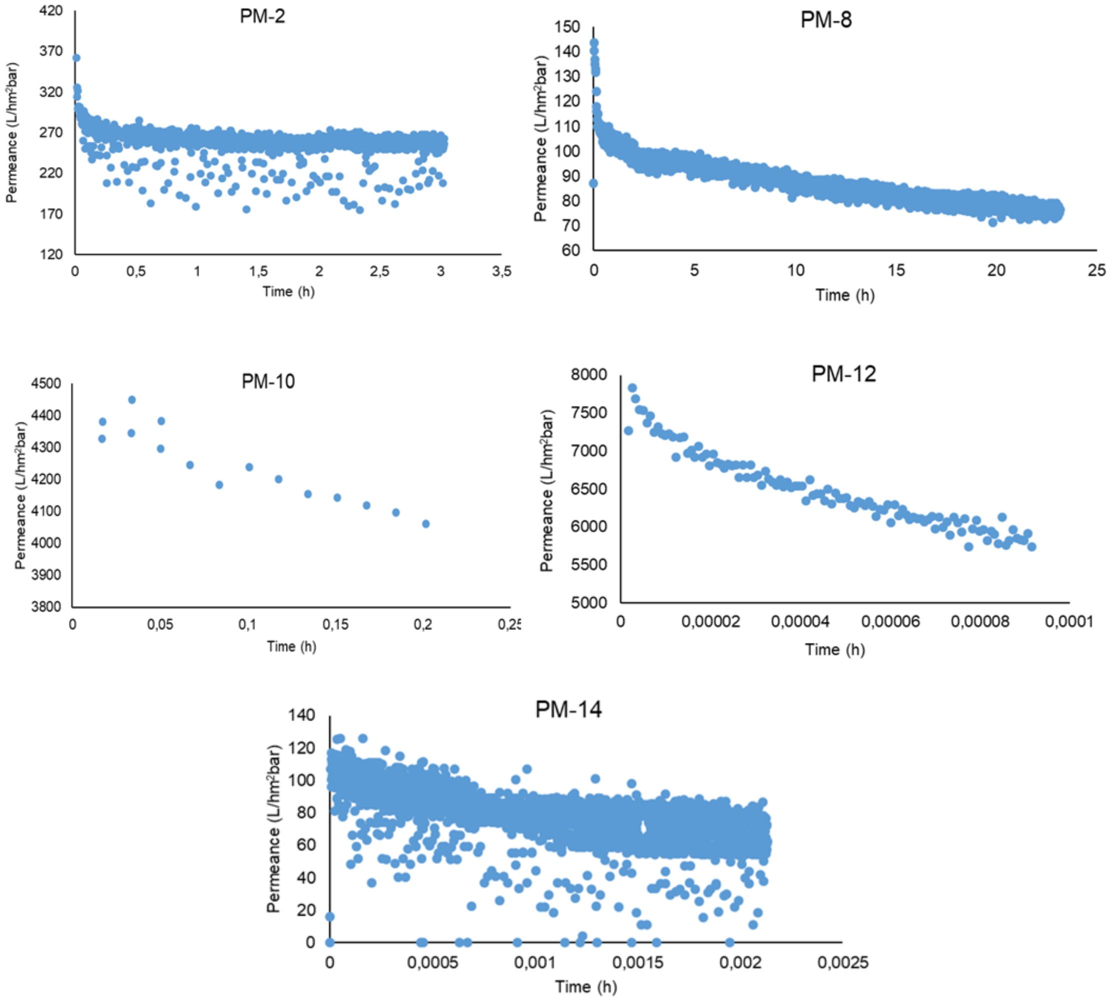

Figure S12: Water flux of membranes

Water flux measurement of PM-1 could not be carried out due to exfoliation of the membrane from the support.
